# Octadecylpropyl Sulfamide Reduces Neurodegeneration and Restores the Memory Deficits Induced by Hypoxia-Ischemia in Mice

**DOI:** 10.3389/fphar.2018.00376

**Published:** 2018-04-19

**Authors:** Elk Kossatz, Daniel Silva-Peña, Juan Suárez, Fernando R. de Fonseca, Rafael Maldonado, Patricia Robledo

**Affiliations:** ^1^Laboratory of Neuropharmacology, Pompeu Fabra University, Barcelona, Spain; ^2^Instituto de Investigación Biomédica de Málaga (IBIMA), UGC Salud Mental, Universidad de Málaga, Hospital Regional Universitario de Málaga, Málaga, Spain; ^3^Departamento de Psicobiología, Facultad de Psicología, Universidad Complutense de Madrid, Madrid, Spain; ^4^Integrative Pharmacology and Systems Neuroscience, IMIM-Hospital del Mar Medical Research Institute, Barcelona, Spain

**Keywords:** hypoxia, ischemia, microglia, memory deficits, neurodegeneration, neuroinflammatory, PPAR-α, endocannabinoid

## Abstract

The PPAR-α agonist, oleoylethanolamide (OEA) has neuroprotective properties in stroke models. However, its rapid degradation represents a limitation for an effective therapeutic approach. In this study, we evaluated the effects of a stable OEA-modeled compound, octadecylpropyl sulfamide (SUL) on the cognitive, behavioral, cellular and molecular alterations associated with hypoxia-ischemia (HI) in mice. Mice subjected to HI were treated with the PPAR-α antagonist GW6471 (GW) (1 mg/kg) followed 15 min later by SUL (3 and 10 mg/kg). Behavioral, motor, and cognitive tests were carried out 24 h and 7 days after the HI. The levels of microglia, reactive astrocytes and neuronal nuclei were studied using immunofluorescence, and the expression of genes related to the *N*-acyl-ethanolamides/endocannabinoid signaling systems was determined by qRT-PCR at the end of the experimental sequence. HI induced brain damage in the ipsilateral hippocampus and cortex, which lead to severe memory impairments, and motor coordination deficits. Significant neuronal loss, increased microglia and reactive astrocytes, and compensatory changes in genes associated with the inflammation/immune and endocannabinoid systems were observed in these brain structures of lesioned mice. SUL reversed the memory and motor deficits, decreased the overexpression of microglia and astrocytes, and reduced neurodegeneration induced by HI. *Cnr1* and *Cnr2* gene expression was modulated by SUL in both sham and HI mice, while *Ppar*α and *Faah* expression was regulated in HI mice. GW completely blocked the beneficial actions of SUL. These findings suggest that treatment with SUL reduces brain damage and the associated motor and memory deficits induced by HI probably by normalizing the changes in neuroinflammation/immune system mediators.

## Introduction

Cerebral ischemia is one of the leading causes of mortality and severe disability worldwide (Mathers et al., 2006; Flynn et al., 2008). This condition is usually caused by a reduction in blood oxygen content, and if the blood flow is not rapidly restored, there is a risk of damage to brain tissues that can lead to severe neurological deficits depending on the duration, location, and size of the insult. In the last years, progress has been achieved in the understanding of the cellular and molecular basis of ischemia pathophysiology (Lo et al., 2003). However, there are no effective therapies at present capable of reducing the long-term consequences derived of ischemia-induced brain damage.

The peroxisome proliferator-activated receptor alpha (PPAR-α) is a nuclear receptor involved in different processes including modulation of cellular differentiation, metabolism of carbohydrates, lipids and proteins, and tumorigenesis (Lee et al., 2003; Marx et al., 2004). A growing amount of evidence suggests that this receptor participates in inflammatory response modulating the expression of chemokines, chemokine receptors and adhesion molecules in endothelial cells, smooth muscle cells, monocytes/macrophages and T cells (Duval et al., 2002; Blanquart et al., 2003), and also increases the expression of antioxidant enzymes, such as superoxide dismutase and catalase (Toyama et al., 2004). In addition, endogenous PPAR-α agonists, such as oleoylethanolamide (OEA) have shown neuroprotective properties on ischemic stroke models through the activation of PPAR-α signaling (Sun et al., 2007; Zhou et al., 2012, 2017; Yang et al., 2015). However, the rapid degradation of the OEA by fatty acid amide hydrolase (FAAH) is known to represent a limitation for the development of an effective therapeutic approach. Modeling OEA analog drugs with high affinity for the PPAR-α might solve this issue and facilitate the development of more stable neuroprotectant drugs.

In the present study, we evaluated the neuroprotective effects of octadecylpropyl sulfamide (SUL), the most potent and stable compound from these new sulfamide derivatives of OEA (Cano et al., 2007), in an adult mouse model of hypoxia-ischemia (HI). SUL was characterized as an effective activator of PPAR-α, with potent hypolipidemic properties, and feeding suppressant effects (Cano et al., 2007; Moreno-Santos et al., 2014). Moreover, SUL showed a neuroprotective effect on neuron cultures exposed to the neurotoxin 6-OHDA, reducing the cell death, and increasing the cell viability (Rodríguez de Fonseca et al., 2012).

Thus, the main goal of this study was to assess the effects of SUL on the cognitive and behavioral, alterations associated with HI brain damage, and to determine whether these effects were mediated by the PPAR-α. To understand the cellular and molecular mechanisms involved in the effects of SUL, we examined neurodegeneration processes, the levels of microglia and reactive astrocytes, as well as, changes in gene expression related to the *N*-acylethanolamides (NAEs)/endocannabinoid signaling systems in different brain areas following the HI insult.

## Experimental Procedures

### Animals

Adult male C57BL/6J mice (8–10 weeks of age) were used in all experiments (Charles River, France). Mice were group-housed and maintained in a temperature (21 ± 1°C) and humidity (65 ± 10%) controlled room with food and water available *ad libitum*. Animal procedures were conducted in accordance with the standard ethical guidelines (EU Directive 2010/63/EU for animal experiments) and approved by the local ethical committee (CEEA, PRBB). All the experiments were performed under blind conditions during the light phase of the dark/light cycle (lights on from 08:00 to 20:00 h).

### Surgery

Hypoxia-ischemia was conducted with some modifications of the originally reported model (Levine, 1960), as previously described (Kossatz et al., 2016). Briefly, mice were anesthetized with isoflurane [a mixture of O_2_ and N_2_O (0.3/0.7 L/min)], a median incision in the neck was performed, and the left common carotid artery was permanently ligated in two locations with 4–0 surgical silk. During the surgery, mice were placed on a heat plate to maintain a constant body temperature of 37°C. One hour after carotid artery occlusion, when the animals had recovered from anesthesia, a hypoxic episode was conducted using a hypoxia chamber (BioSpherix, Parish, NY, United States) consisting of a constant flow of 10% oxygen for 60 min. The temperature of the mice (37°C) was maintained inside the chamber via heating devices placed beneath the animal cage. Sham-operated animals received the same procedure except for the common carotid artery ligation and hypoxia. A mouse rectal probe was used to measure the body temperature before and after surgery, and before and after hypoxia. An analgesic (meloxicam, 1 mg/kg) was administered before the surgery and then once daily during 3 days. After HI, mice were managed according to the treatment protocol.

### Drugs and Treatments

The SUL was synthesized in the Laboratorio de Medicina Regenerativa of Hospital Regional Universitario de Málaga, as previously described (Cano et al., 2007). The PPAR-α antagonist GW6471 (GW) was purchased from Tocris Bioscience (Madrid, Spain) and diluted in a vehicle (VEH) solution prepared with 10% DMSO and 90% saline. SUL was diluted in 5% Tween 80 and 95% saline. SUL and GW were administered intraperitoneally (i.p.) in a volume of 20 and 10 ml/kg of body weight, respectively. SUL was administered at doses reported previously to produce overt effects on feeding behavior (Cano et al., 2007).

In Experiment 1, to determine the effects of SUL on the cognitive deficits induced by HI, mice received an acute administration of SUL at 3 (SUL3) and 10 mg/kg (SUL10), or VEH 15 min after the HI insult. After treatment, mice were placed into individual chambers and allowed free access to food and water. The behavioral test was conducted 7 days after surgery, and animals were sacrificed at the end of testing to analyze the extent of the lesion.

In Experiment 2, to assess whether the effects of SUL were mediated through the PPAR-α, mice were treated with the optimum dose of SUL (10 mg/kg) found in experiment 1, and with the PPAR-α antagonist, GW at the dose of 1 mg/kg and tested in the behavioral paradigms. Mice were assigned to different treatment groups: VEH/VEH, VEH/SUL10, GW/SUL10, or GW/VEH, and received one dose of GW (1 mg/kg, i.p.) or VEH immediately after the HI episode. SUL10 (10 mg/kg, i.p.) or VEH were injected once 15 min after the antagonist treatment (GW or VEH). After drug administration, mice were placed into individual chambers and allowed free access to food and water. The behavioral tests were conducted 24 h and 7 days after surgery, and the animals were sacrificed at the end of testing for subsequent biochemical and molecular analysis of the brain.

### Behavioral Tests

In experiment 1, the cognitive alterations were evaluated 7 days after HI using the novel object recognition test (NOR). In experiment 2, the behavioral and motor alterations associated with HI-induced brain damage were evaluated 24 h and 7 days post HI, while the NOR test was carried out only 7 days after the injury. The sequence of tests performed on day 7 was as follows: First mice were tested in the Irwin test, then in the rotarod, the beam-walking and the open field tests. Finally, the NOR training was carried out, followed by the NOR test 3 h later (see Supplementary Figure S1 for time-line). All the tests were conducted as previously described (Kossatz et al., 2016). Irwin test: To evaluate subjective signs of behavioral dysfunction produced by HI, a modified Irwin test (Irwin, 1968) was conducted. Some symptoms were evaluated by their presence or absence (loss of balance, loss of traction, ptosis and motor incoordination) on a scale from 0 to 1 (absence = 0, presence = 1). Other symptoms (abnormal gait, piloerection, low reactivity to touch) were rated on a scale from 0 to 6 according to the severity (no symptoms = 0, severe symptoms = 6), and neurological deficits were rated on a scale from 0 to 2 (no deficit = 0, forelimb weakness and torso turning to the affected side when held by the tail = 1 or circling to affected side = 2). The final score was expressed by the sum of all symptoms observed in each animal.

Open field test: Mice were placed in an experimental arena (Plexiglas box, 90 × 70) to evaluate locomotor activity, and the total distance traveled was determined by the sum of squares crossed (units) during a 5 min period.

Rotarod test: Mice were trained for 2 days before HI in the accelerating rotarod (Panlab) to evaluate motor coordination. The first training consisted of 60 s at 6 rpm, and the second training was 60 s at 8 rpm. On the test day, mice were tested over five trials using the rotarod in the acceleration mode from 4 to 20 rpm until the animal fell from the rod. The latency to fall off the rod on every trial was determined, and the mean of the five trials was used in the data analysis.

Beam-walking test: The ability of the mice to remain upright and to walk on an elevated beam was tested to evaluate the motor coordination and balance. The beams consisted of 1-m long strips of wood with 20 or 6 mm round cross sections. The beams were placed horizontally, 40 cm above the floor, with one end mounted on a support and the other end attached to the home cage of the animal as the escape box. Mice were trained for 2 days before HI, which consisted of two trials with the animal traversing the 20 mm beam. On the test day, mice were evaluated in two trials using the narrower 6 mm beam. The latency to traverse the beam was determined, and the mean scores of the two trials were used in the data analysis.

Novel object recognition test (NOR): We used the NOR test for consistency purposes since in a previous study we observed object recognition memory deficits by similar HI-induced brain damage in the hippocampus and the adjacent entorhinal, perirhinal, and parahippocampal cortices (Kossatz et al., 2016), brain structures involved in this kind of memory (Zola-Morgan and Squire, 1993; Zola et al., 2000). A black Plexiglas “V” maze with two corridors (30 cm long × 4.5 cm wide, and 15 cm high walls) set at a 90° angle (Panlab, Barcelona, Spain) was used to evaluate short-term (3 h) memory deficits. On day 6 mice were habituated to the empty maze for 9 min. The next day (day 7), mice were introduced in the maze for 9 min, and were allowed to explore two identical objects placed on each arm of the maze (training session). The memory test lasting 9 min was carried out 3 h later, where one of the familiar objects was replaced by a novel object. The total time spent exploring each object (novel and familiar) was recorded. A discrimination index (DI) was calculated as the difference between the time spent exploring either the novel (Tn) or familiar (Tf) object divided by the total time exploring both objects [DI = (Tn - Tf)/(Tn + Tf)]. A higher discrimination index indicates better memory retention. Mice were excluded from the test if the exploration of each object was less than 2 s, or if the total exploration time of both objects was less than 10 s.

### Histological Analysis and Immunofluorescence

Seven days after the HI procedure, mice were deeply anesthetized by intraperitoneal injection (0.2 mL per 10 g body weight) of a mixture of ketamine (100 mg/kg) and xylazine (20 mg/kg), and intracardially perfused with 4% paraformaldehyde in 0.1 M phosphate buffer (pH 7.5), with a peristaltic pump. Brains were removed and post-fixed overnight at 4°C in the same fixative solution and cryoprotected in a solution of 30% sucrose at 4°C during 24 h. Brain sections were sliced at 30 μm with a microtome from 1.98 to 1.34, 1.18 to 0.38, -0.22 to -0.88, -1.46 to -2.06, and -2.30 to -2.96 relative to Bregma (Paxinos and Franklin, 2001) and stored at 4°C in a solution containing 5% sucrose and 0.02% sodium azide in 0.1 M phosphate buffer.

To determine the extent of the lesion, a standard Cresyl violet staining protocol was used to identify the infarct area (loss of Nissl staining) in the HI ipsilateral hemisphere. The stained sections were analyzed at 1× (zoom factor 0.8×) using an Olympus MVX10 Macro Zoom Microscope equipped with Olympus DP71 digital camera. The ischemic lesion was delimited using the ImageJ software (NIH). For each mouse, the lesioned area was calculated in five brain sections, and the sum in pixels was transformed to μm^2^ and expressed as a percentage of the contralateral hemisphere to correct for possible edema in the ipsilateral hemisphere (Rousselet et al., 2012).

To determine the expression levels of glial fibrillary acid protein (GFAP), Iba-1 and NeuN in the ipsilateral cortex and hippocampus, single-immunofluorescence studies were carried as previously described (Cutando et al., 2013). Free-floating slices were placed in 0.3% Triton X-100, 10% donkey serum or goat serum (Sigma) in PB and incubated overnight at 4°C with the following primary antibodies: rabbit anti-GFAP (1:500, Dako; cat. #Z0334), rabbit anti-Iba-1 (1:500, Wako; cat. no. 019-19741) and mouse anti-NeuN (1:500, Millipore; cat. no. MAB377). The following day, sections were incubated for 2 h at room temperature with the following secondary antibodies: Alexa 488 donkey anti–rabbit-Cy2 and Alexa 555 goat anti-mouse-Cy3 (1:500; Invitrogen/Molecular Probes). Confocal images were obtained using a Leica TSC SP8 confocal microscope at 20× with a 1.5 zoom increase. To quantify GFAP, Iba-1 and NeuN expression, six images of stained sections (from -1.46 to -2.06) per animal were analyzed for each experimental group using ImageJ software. Positive cells in each structure were averaged and compared between experimental groups.

### RNA Isolation and RT-qPCR Analysis

To assess changes in the expression of different genes related to the endocannabinoid and neuroimmune systems in the ipsilateral motor cortex and hippocampus of sham and lesioned mice, qPCR analysis was carried out in the following treatment groups: VEH/VEH (*n* = 6 HI mice; *n* = 6 sham-operated), VEH/SUL10 (*n* = 6 HI mice; *n* = 6 sham-operated), GW/VEH (*n* = 6 HI mice; *n* = 6 sham-operated), GW/SUL10 (*n* = 6 HI mice; *n* = 6 sham-operated). Seven days after behavioral testing, mice were euthanized by cervical dislocation. Fresh brains were harvested and sliced, using a coronal brain matrix (1 mm), into three parallel series relative to Bregma (Paxinos and Franklin, 2001): from 2.34 to 0.26 to obtain the motor cortex, from -1.22 to -2.30 to obtain the dorsal hippocampus, and from -2.30 to -3.40 to obtain the ventral hippocampus. The motor cortex and dorsal hippocampus were stored at -80°C for posterior PCR analysis. The ventral hippocampus was sliced at 20 μm with a cryostat, mounted onto gelatin coated slides and stained with Cresyl violet to distinguish lesioned from the non-lesioned animals. We performed reverse transcription (RT) and quantitative real-time polymerase chain reaction (qPCR) as described previously (Ramírez-López et al., 2016) using specific sets of primer probes [*ActB*: Mm02619580-g1, Amplicon length: 143; *GusB*: Mm01197698-m1, Amplicon length: 71; *Gapdh*: Mm99999915-g1, Amplicon length: 109; *Gfap*: Mm01253033-m1, Amplicon length: 75; *Iba-1*: Mm00479862-g1, Amplicon length: 82; *Cox2* (Ptgs2): Mm00478374-m1, Amplicon length 80; *Fcgr2b*: Mm00438875-m1, Amplicon length: 77; *Mrc1*: Mm01329362-m1, Amplicon length: 147; *Cnr1*: Mm01212171-s1, Amplicon length: 66; *Cnr2*: Mm02620087-s1, Amplicon length: 171; *Napepld*: Mm00724596-m1, Amplicon length: 85; *Faah*: Mm00515684-m1, Amplicon length: 62; *Ppar*α: Mm00440939-m1, Amplicon length: 74; Life Technologies]. Briefly, motor cortex and hippocampus were homogenized on ice and RNA was extracted following Trizol^®^ method according to the manufacturer’s instruction (Gibco BRL Life Technologies, Baltimore, MD, United States). RNA samples were isolated with RNeasy minelute clean-up-kit including digestion with DNase I column (Qiagen, Hilden, Germany). After RT reaction from 1 μg of mRNA, qPCR was performed in a CFX96TM Real-Time PCR Detection System (Bio-Rad, Hercules, CA, United States) and the FAM dye label format for the TaqMan^®^ Gene Expression Assays (Applied Biosystem, Carlsbad, CA, United States). Melting curve analysis was performed to ensure that only a single product was amplified. After analyzing several control genes, values obtained from the brain samples were normalized in relation to *Gapdh* gene expression levels.

### Statistical Analyzes

The effects of treatments on the mortality ratio and the percent of mice with/without lesions after HI were analyzed using a χ-square non-parametrical test. The extent of the lesion was analyzed using one- or two-way ANOVAs. The behavioral and immunofluorescence data were analyzed using two- or three-way ANOVAs followed by the Fisher’s LSD *post hoc* test for multiple comparisons, when appropriate (Statistica version 6). The RT-qPCR data were analyzed using a three-way ANOVA followed by the Bonferroni *post hoc* test when appropriate (GraphPad Prism version 5.04). A *p*-value below 0.05 was considered statistically significant.

## Results

### SUL Prevents the Cognitive Alterations Induced by HI

In experiment 1, no significant differences in mortality or in the percentage of lesioned animals were observed between groups (**Table 1**). In lesioned mice, brain damage was observed in the ipsilateral hippocampus, an extensive part of the cortex, the striatum and the amygdala. The contralateral hemisphere remained intact, and no overt signs of brain injury were observed in sham animals on either hemisphere. To reduce variability and improve data homogeneity, lesioned mice that showed brain damage smaller than 6% with respect to the contralateral hemisphere were excluded from the histological and NOR analysis.

**Table 1:** Effects of octadecylpropyl sulfamide on mortality, variability (lesioned and non-lesioned mice), and extent of lesion following HI.

	VEH	SUL3	SUL10
Mortality	3.3%	0	0
Lesioned mice	46.7%	37.5%	35%
Non-lesioned mice	50%	62.5%	65%
Total *n*	30	40	20

Extent of lesion (% CH)	23.63%	20.56%	16.80%
Total *n*^∗^	9	7	6

*VEH, vehicle; SUL3, octadecylpropyl sulfamide (3 mg/kg); SUL10, octadecylpropyl sulfamide (10 mg/kg); CH, contralateral hemisphere. ^∗^Animals that showed more than 6% of lesioned area.*

**FIGURE 1:**
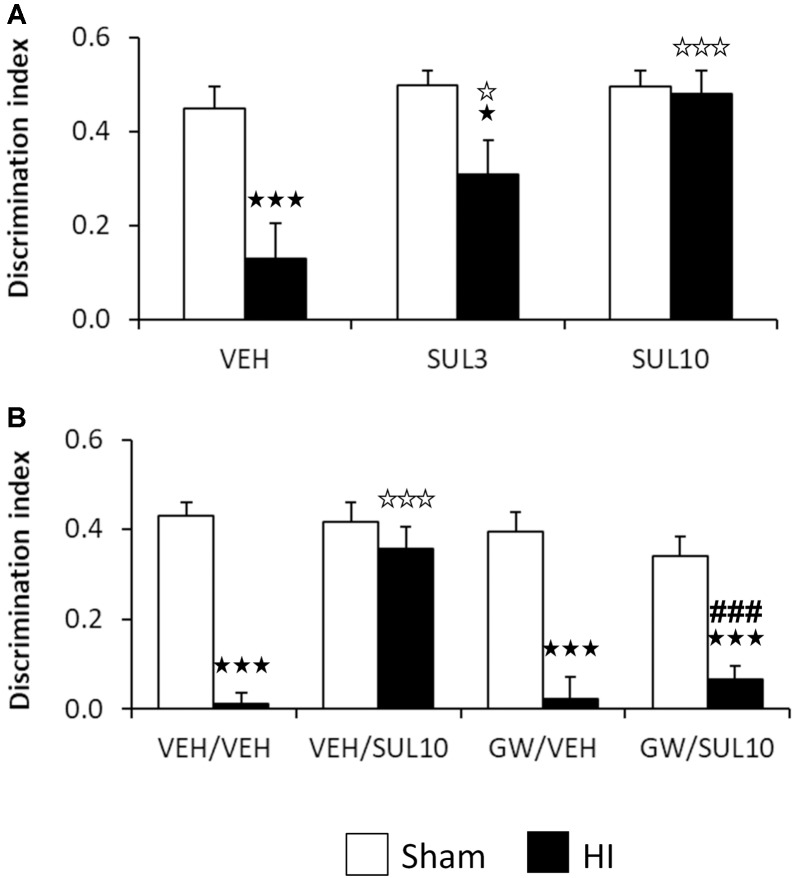
Effects of an acute administration of octadecylpropyl sulfamide (SUL3, 3 mg/kg; SUL10, 10 mg/kg), vehicle (VEH) and GW6471 (GW, 1 mg/kg) on the memory deficits induced by HI. **(A)** Discrimination index (DI) at 7 days post-HI in the novel object recognition test in VEH (*n* = 9 HI mice; *n* = 7 sham-operated), SUL3 (*n* = 7 HI mice; *n* = 8 sham-operated) and SUL10 (*n* = 6 HI mice; *n* = 7 sham-operated) treatments groups. A significant decrease in memory was observed in the HI group treated with VEH with respect to sham-operated mice (*p* < 0.001). This effect was completely reversed by SUL10 (*p* < 0.001) and partially by SUL3 (*p* < 0.05). **(B)** Discrimination index at 7 days post-HI in the novel object recognition test in VEH/VEH (*n* = 20 HI mice; *n* = 14 sham-operated), VEH/SUL10 (*n* = 16 HI mice; *n* = 13 sham-operated), GW/VEH (*n* = 12 HI mice; *n* = 12 sham-operated), and GW/SUL10 (*n* = 11 HI mice; *n* = 13 sham-operated) treatments groups. Significant memory deficits were observed in HI mice treated with VEH/VEH with respect to sham-operated mice (*p* < 0.001). This effect was reversed in the group treated with VEH/SUL10 (*p* < 0.001), and GW/VEH significantly blocked the beneficial effects of SUL10 (*p* < 0.001). The administration of GW/VEH did not significantly modify the DI in sham-operated or HI mice with respect to VEH/VEH treatment. Data are expressed as mean + SEM. ^
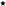
^*p* < 0.05, ^
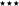
^*p* < 0.001 vs. sham-operated mice of the same group; ^
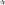
^*p* < 0.05, ^
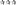
^*p* < 0.001 vs. VEH/VEH HI mice; ^###^*p* < 0.001 vs. VEH/SUL10 HI mice.

The dose-response of SUL on object recognition memory was evaluated 7 days after the HI insult in the NOR test in sham and HI mice receiving SUL3, SUL10 or VEH (**Figure 1A**). No differences were observed between groups with respect to the initial exploration of the maze, indicating a lack of preference bias for the right and left arm. For the test session, two-way ANOVA revealed significant effects of treatment [*F*(2,37) = 6.5, *p* < 0.01], lesion [*F*(1,37) = 15.4, *p* < 0.001], and interaction between factors [*F*(2,37) = 3.8, *p* < 0.05]. *Post hoc* analyses showed significant memory deficits in the HI group treated with VEH (*p* < 0.001) with respect to sham-operated animals. This effect was completely reversed by SUL10 (*p* < 0.001), and only partially by SUL3 (*p* < 0.05). Therefore, we used the dose of 10 mg/kg in subsequent experiments.

### The PPAR-α Mediates the Beneficial Effects of SUL on Cognitive Processing

In experiment 2, no significant differences were observed in terms of mortality or percentage of lesioned mice in the different treatment groups (**Table 2**). Lesioned mice showed brain damage in similar areas than in experiment 1.

**Table 2:** Effects of octadecylpropyl sulfamide and GW6471 on mortality, variability (lesioned and non-lesioned mice), and extent of lesion following HI.

	VEH/VEH	VEH/SUL10	GW/VEH	GW/SUL10
Mortality	5.3%	0	8%	4%
Lesioned mice	42.1%	23.6%	34%	24%
Non-lesioned mice	52.6%	76.4%	58%	72%
Total *n*	76	72	50	50

Extent of lesion (% CH)	19.59%	14.62%	29.80%	17.39%
Total *n*^∗^	14	10	6	5

*VEH, vehicle; SUL10, octadecylpropyl sulfamide (10 mg/kg); GW, GW6471 (1 mg/kg). CH, contralateral hemisphere. ^∗^Animals that showed more than 6% of lesioned area.*

The involvement of the PPAR-α on the cognitive effects of SUL10 in HI mice was evaluated 7 days after HI (**Figure 1B**). Three-way ANOVA revealed significant effects of antagonist [*F*(1,103) = 11.7, *p* < 0.001], treatment [*F*(1,103) = 8.0, *p* < 0.01], and lesion [*F*(1,103) = 98.7, *p* < 0.001]. Significant interactions were observed between antagonist and treatment [*F*(1,103) = 9.1, *p* < 0.01], treatment and lesion [*F*(1,103) = 16.3, *p* < 0.001], and between antagonist, treatment and lesion [*F*(1,103) = 5.3, *p* < 0.05]. Subsequent analyses revealed significant memory deficits in HI mice treated with VEH/VEH with respect to sham-operated mice (*p* < 0.001). This effect was reversed in the group treated with VEH/SUL10 (*p* < 0.001), and GW/VEH significantly blocked the beneficial effects of VEH/SUL10 (*p* < 0.001). The administration of GW/VEH did not significantly modify the DI in sham-operated or HI mice with respect to VEH/VEH treatment. These data indicate that SUL10 prevents the memory deficits caused by HI through a mechanism involving the PPAR-α.

### Effects of SUL and GW on Behavioral, Motor Coordination, Balance and Locomotor Activity Alterations Induced by HI

**FIGURE 2:**
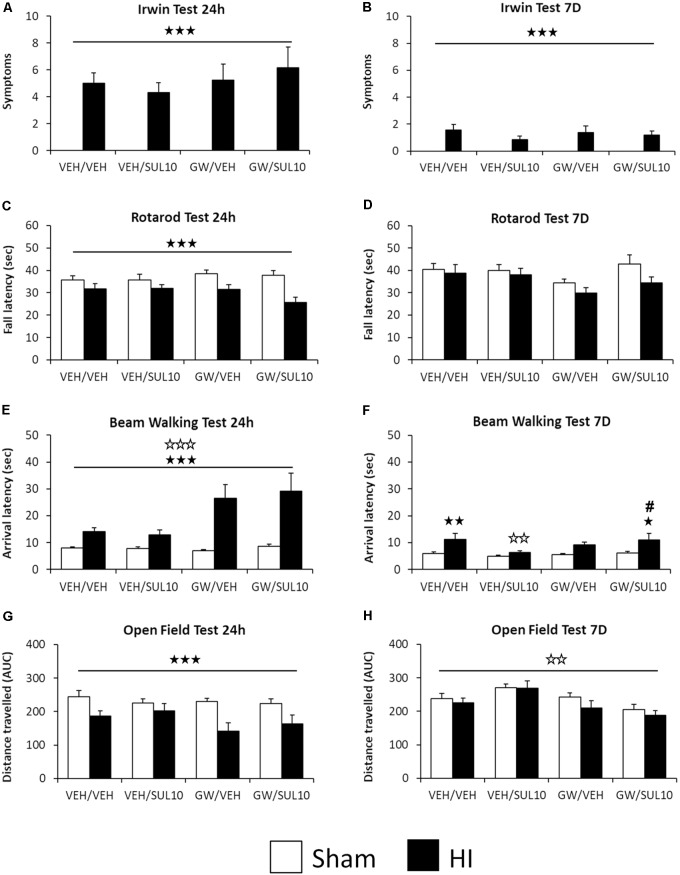
Effects of an acute administration of octadecylpropyl sulfamide (SUL10, 10 mg/kg) and GW6471 (GW, 1 mg/kg) on the behavioral deficits induced by HI at 24 h and 7 days in VEH/VEH (*n* = 21 HI mice; *n* = 14 sham-operated), VEH/SUL10 (*n* = 16 HI mice; *n* = 14 sham-operated), GW/VEH (*n* = 12 HI mice; *n* = 12 sham-operated), GW/SUL10 (*n* = 11 HI mice; *n* = 14 sham-operated) treatment groups. In the Irwin test **(A,B)**, the rotarod test **(C,D)**, and the open field test **(G,H)** mostly simple effects and no interactions between factors were observed. In the beam walking test **(E,F)**, interactions were observed only 7 days after HI **(F)**, *post hoc* analysis revealed significant motor coordination deficits in HI mice treated with VEH/VEH (*p* < 0.01) and GW/SUL10 with respect to sham-operated animals (*p* < 0.05). Treatment with VEH/SUL10 significantly decreased the arrival latency in HI mice as compared to VEH/VEH administration (*p* < 0.01), and GW/VEH reversed this effect (*p* < 0.05). The administration of GW/VEH did not significantly modify the arrival latency in sham-operated or HI mice with respect to VEH/VEH treatment. All data expressed as mean + SEM. ^
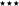
^*p* < 0.001 (lesion effect), ^
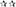
^*p* < 0.01, ^
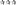
^*p* < 0.001 (antagonist effect). In 2F: ^
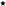
^*p* < 0.05, ^
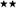
^*p* < 0.01 vs. sham-operated mice of the same group; ^
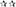
^*p* < 0.01 vs. VEH/VEH HI mice; ^#^*p* < 0.05 vs. VEH/SUL10 HI mice.

In the Irwin test, statistical analysis for the number of symptoms observed revealed significant effects of lesion 24 h [*F*(1,107) = 96.7, *p* < 0.001] and 7 days [*F*(1,107) = 34.1, *p* < 0.001] following HI (**Figures 2A,B**). In the rotarod test, the analysis of fall latency showed a significant effect of lesion [*F*(1,107) = 18.7, *p* < 0.001] 24 h following HI only (**Figure 2C**), while at 7 days, no significant main effects or interactions were revealed (**Figure 2D**).

In the beam walking test 24 h following HI, the analysis of arrival latency revealed significant effects of antagonist [*F*(1,107) = 14.6, *p* < 0.001] and lesion [*F*(1,107) = 47.5, *p* < 0.001] (**Figure 2E**). At 7 days, significant effects of lesion [*F*(1,107) = 14.4, *p* < 0.001] and interaction between antagonist and treatment were revealed [*F*(1,107) = 4.2, *p* < 0.05]. *Post hoc* comparisons revealed significant motor coordination deficits in HI mice treated with VEH/VEH (*p* < 0.01) and GW/SUL10 with respect to sham-operated animals (*p* < 0.05). Treatment with VEH/SUL10 significantly decreased the arrival latency in HI mice as compared to VEH/VEH administration (*p* < 0.01), and GW reversed this effect (*p* < 0.05). The administration of GW/VEH did not significantly modify the arrival latency in sham-operated or HI mice with respect to VEH/VEH treatment (**Figure 2F**). These results indicate that SUL10 has beneficial effects on balance and motor coordination deficits by acting on the PPAR-α.

In the open field test, statistical analysis for distance traveled revealed a significant effect of lesion [*F*(1,107) = 19.1, *p* < 0.001], 24 h (**Figure 2G**), and a significant effect of antagonist [*F*(1,107) = 10.7, *p* < 0.01] 7 days following HI (**Figure 2H**).

### SUL Reverses the Increase in Microglia Following HI Through the PPAR-α

**FIGURE 3:**
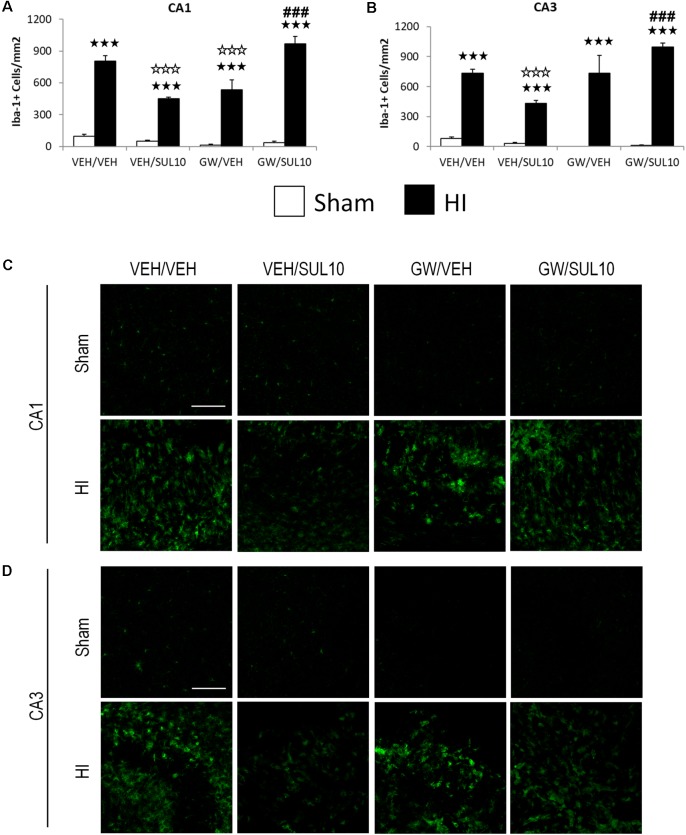
Effects of an acute administration of octadecylpropyl sulfamide (SUL10, 10 mg/kg) and GW6471 (GW, 1 mg/kg) on the number of Iba-1 positive cells induced by HI in the ipsilateral hippocampal areas. **(A,C)** In the CA1, a significant effect of lesion [*F*(1,50) = 402.4, *p* < 0.001], and interactions between antagonist and treatment [*F*(1,50) = 44.9, *p* < 0.001], antagonist and lesion [*F*(1,50) = 7.1, *p* < 0.01], and between antagonist, treatment and lesion [*F*(1,50) = 31.1, *p* < 0.001] were observed. **(B,D)** In the CA3, significant effects of lesion [*F*(1,50) = 270.2, *p* < 0.001], and interactions between antagonist and treatment [*F*(1,50) = 13.8, *p* < 0.001], antagonist and lesion [*F*(1,50) = 15.7, *p* < 0.001], and between antagonist, treatment and lesion [*F*(1,50) = 9.1, *p* < 0.01] were observed. Data represent mean + SEM cells per mm^2^ in VEH/VEH (*n* = 11 HI mice; *n* = 7 sham-operated), VEH/SUL10 (*n* = 9 HI mice; *n* = 8 sham-operated), GW/VEH (*n* = 6 HI mice; *n* = 6 sham-operated), GW/SUL10 (*n* = 5 HI mice; *n* = 7 sham-operated) treatment groups. The panels show representative images of Iba-1 expression in CA1 **(C)** and CA3 **(D)**. Scale bar = 100 μm. ^
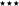
^*p* < 0.001 vs. sham-operated mice of the same group; ^
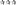
^*p* < 0.001 vs. VEH/VEH HI mice; ^###^*p* < 0.001 vs. VEH/SUL10 HI mice.

In the CA1 (**Figures 3A,C**), the expression of Iba-1 was increased in HI mice treated with VEH/VEH (*p* < 0.001), VEH/SUL10 (*p* < 0.001), GW/VEH (*p* < 0.001), and GW/SUL10 (*p* < 0.001) with respect to sham-operated mice. Notably, SUL10 significantly reversed the increase in microglia expression observed in HI mice treated with VEH (*p* < 0.001), and GW significantly blocked this effect (*p* < 0.001).

Similar results were obtained in the CA3 area (**Figures 3B,D**). The expression of Iba-1 was increased in HI mice treated with VEH/VEH (*p* < 0.001), VEH/SUL10 (*p* < 0.001), GW/VEH (*p* < 0.001), and GW/SUL10 (*p* < 0.001) with respect to sham-operated animals. SUL10 significantly decreased the overexpression of microglia observed in the HI mice treated with VEH (*p* < 0.001), and GW significantly blocked this effect (*p* < 0.001).

**FIGURE 4:**
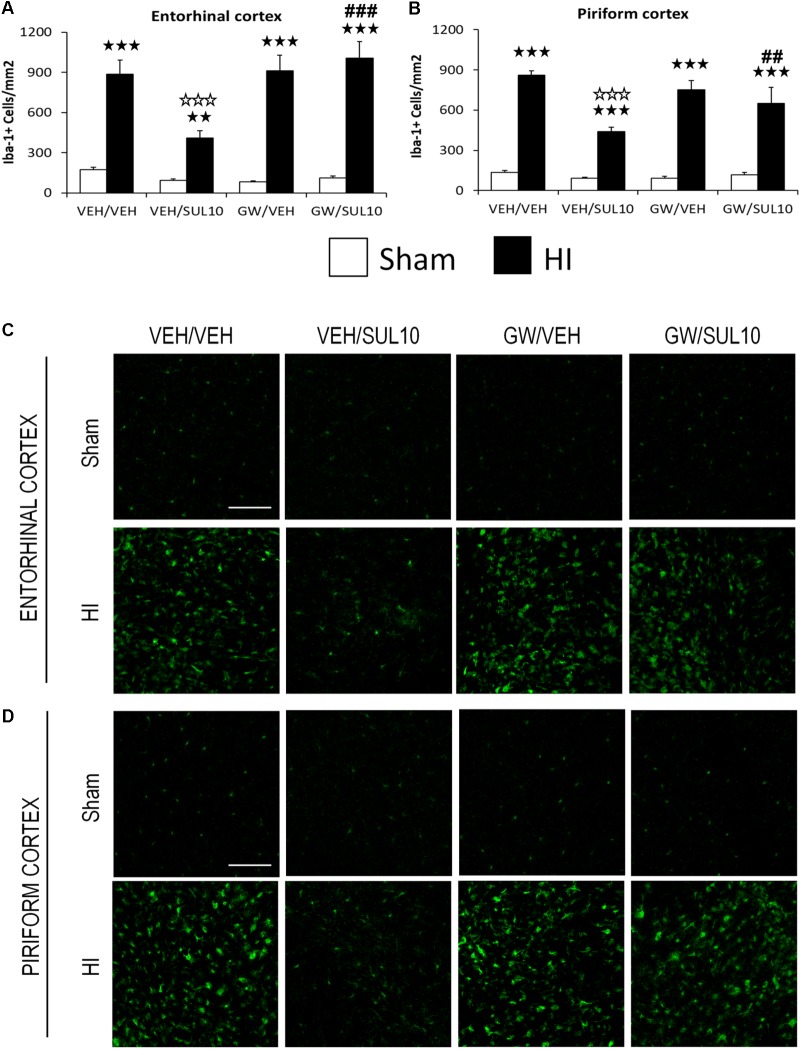
Effects of an acute administration of octadecylpropyl sulfamide (SUL10, 10 mg/kg) and GW6471 (GW, 1 mg/kg) on the number of Iba-1 positive cells induced by HI in ipsilateral cortical areas. **(A,C)** In the entorhinal cortex significant effects of antagonist [*F*(1,51) = 6.1, *p* < 0.05], lesion [*F*(1,51) = 154.2, *p* < 0.001], and interactions between antagonist and treatment [*F*(1,51) = 9.4, *p* < 0.01], antagonist and lesion [*F*(1,51) = 9.7, *p* < 0.01], and between antagonist, treatment and lesion [*F*(1,51) = 4.3, *p* < 0.05] were observed. **(B,D)** In the piriform cortex, significant effects of treatment [*F*(1,51) = 19.5, *p* < 0.001], lesion [*F*(1,51) = 343.4, *p* < 0.001], and interactions between antagonist and treatment [*F*(1,51) = 10.1, *p* < 0.01], treatment and lesion [*F*(1,51) = 16.9, *p* < 0.001], and between antagonist, treatment and lesion [*F*(1,51) = 4.2, *p* < 0.05] were observed. Data represent mean + SEM cells per mm^2^ in VEH/VEH (*n* = 11 HI mice; *n* = 7 sham-operated), VEH/SUL10 (*n* = 9 HI mice; *n* = 8 sham-operated), GW/VEH (*n* = 6 HI mice; *n* = 6 sham-operated), GW/SUL10 (*n* = 5 HI mice; *n* = 7 sham-operated) treatment groups. The panels show representative images of Iba-1 expression in entorhinal cortex **(C)** and piriform cortex **(D)**. Scale bar = 100 μm. ^
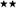
^*p* < 0.01, ^
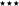
^*p* < 0.001 vs. sham-operated mice of the same group; ^
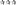
^*p* < 0.001 vs. VEH/VEH HI mice; ^##^*p* < 0.01, ^###^*p* < 0.001 vs. VEH/SUL10 HI mice.

In the entorhinal cortex (**Figures 4A,C**), a significant increase in the expression of Iba-1 in HI mice treated with VEH/VEH (*p* < 0.001), VEH/SUL10 (*p* < 0.01), GW/VEH (*p* < 0.001), and GW/SUL10 (*p* < 0.001) with respect to sham-operated mice was observed. SUL10 significantly reversed the increase in microglia expression observed in HI mice treated with VEH (*p* < 0.001), and GW significantly blocked this effect (*p* < 0.001).

In the piriform cortex (**Figures 4B,D**), the expression of Iba-1 was increased in HI mice treated with VEH/VEH (*p* < 0.001), VEH/SUL10 (*p* < 0.001), GW/VEH (*p* < 0.001), and GW/SUL10 (*p* < 0.001) with respect to sham-operated animals. SUL10 significantly reversed the increase in microglia expression observed in HI mice treated with VEH (*p* < 0.001), and GW significantly blocked this effect (*p* < 0.01).

In the dentate gyrus (Supplementary Figures S2A,C), a significant effect of lesion [*F*(1,50) = 83.2, *p* < 0.001], was found, and in the somatosensory cortex (Supplementary Figures S2B,D), significant effects of antagonist [*F*(1,51) = 13.1, *p* < 0.001], and lesion [*F*(1,51) = 93.3, *p* < 0.001] were revealed.

### SUL Reverses the Overexpression of Reactive Astrocytes Following HI Through the PPAR-α

**FIGURE 5:**
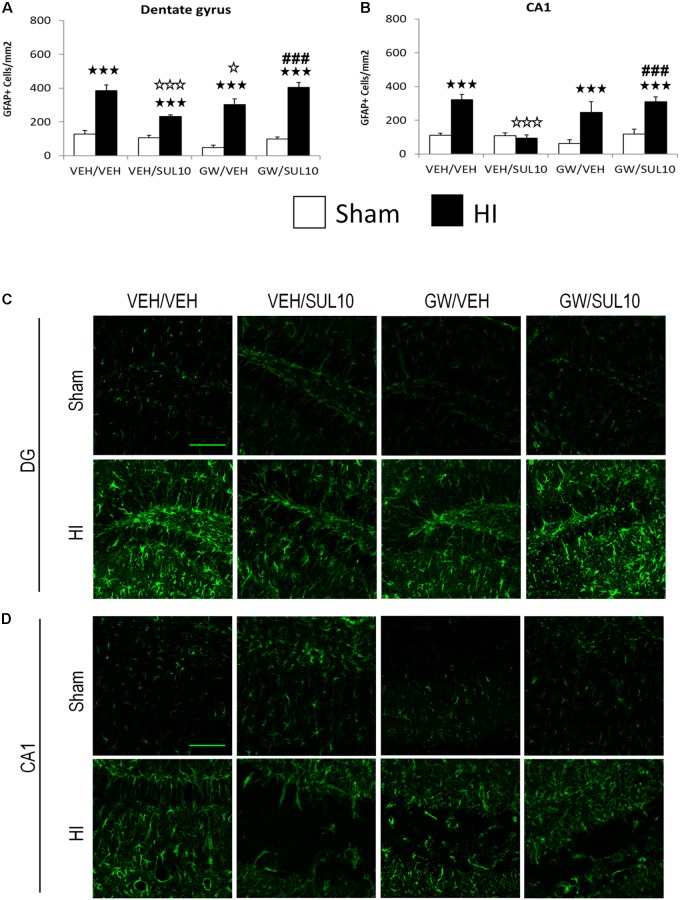
Effects of an acute administration of octadecylpropyl sulfamide (SUL10, 10 mg/kg) and GW6471 (GW, 1 mg/kg) on the number of GFAP positive cells induced by HI in the dentate gyrus and CA1 area of the hippocampus. **(A,C)** In the dentate gyrus, a significant effect of lesion [*F*(1,51) = 186.1, *p* < 0.001], and interactions between antagonist and treatment [*F*(1,51) = 21.9, *p* < 0.001], antagonist and lesion [*F*(1,51) = 6.5, *p* < 0.05], and between antagonist, treatment and lesion [*F*(1,51) = 7.0, *p* < 0.05] were observed. **(B,D)** In the CA1, significant effects of lesion [*F*(1,51) = 43.0, *p* < 0.001], and interactions between antagonist and treatment [*F*(1,51) = 16.1, *p* < 0.001], antagonist and lesion [*F*(1,51) = 4.3, *p* < 0.05], treatment and lesion [*F*(1,51) = 6.2, *p* < 0.05], and between antagonist, treatment and lesion [*F*(1,51) = 6.8, *p* < 0.05] were observed. Data represent mean + SEM cells per mm^2^ in VEH/VEH (*n* = 10 HI mice; *n* = 7 sham-operated), VEH/SUL10 (*n* = 9 HI mice; *n* = 8 sham-operated), GW/VEH (*n* = 6 HI mice; *n* = 7 sham-operated), GW/SUL10 (*n* = 5 HI mice; *n* = 7 sham-operated) treatment groups. The panels show representative images of GFAP expression in the dentate gyrus **(C)** and CA1 **(D)**. Scale bar = 100 μm. ^
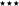
^*p* < 0.001 vs. sham-operated mice of the same group; ^
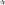
^*p* < 0.05, ^
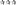
^*p* < 0.001 vs. VEH/VEH HI mice; ^###^*p* < 0.001 vs. VEH/SUL10 HI mice.

In the dentate gyrus (**Figures 5A,C**), the expression of GFAP was increased in HI mice treated with VEH/VEH (*p* < 0.001), VEH/SUL10 (*p* < 0.001), GW/VEH (*p* < 0.001), and GW/SUL10 (*p* < 0.001) with respect to sham-operated mice. Notably, SUL10 significantly reversed the increase in astrocyte expression observed in HI mice treated with VEH (*p* < 0.001), and GW significantly blocked this effect (*p* < 0.001).

Similar results were obtained in the CA1 area (**Figures 5B,D**). The expression of GFAP was increased in HI mice treated with VEH/VEH (*p* < 0.001), GW/VEH (*p* < 0.001), and GW/SUL10 (*p* < 0.001) with respect to sham-operated animals. SUL10 completely reversed the overexpression of astrocytes observed in the HI mice treated with VEH (*p* < 0.001), and GW significantly blocked this effect (*p* < 0.001).

**FIGURE 6:**
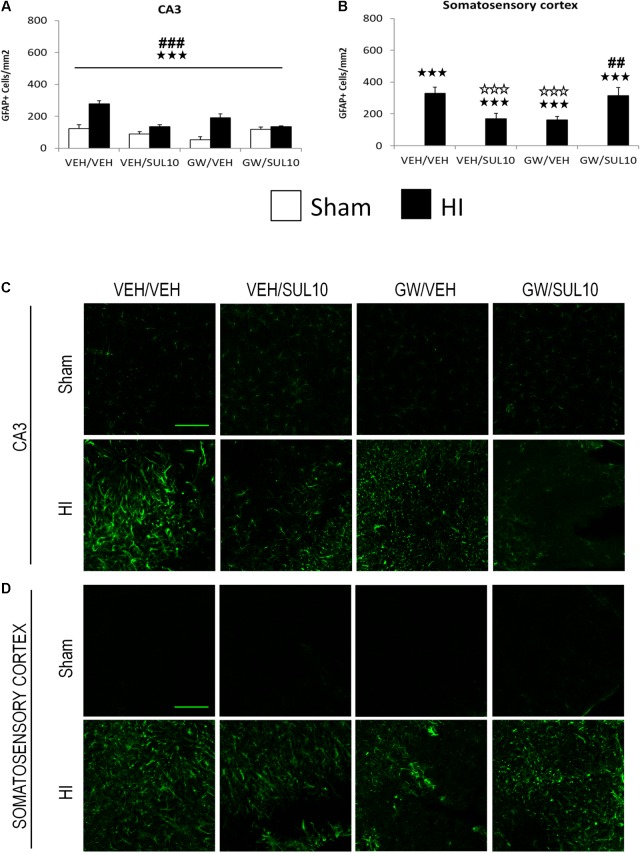
Effects of an acute administration of octadecylpropyl sulfamide (SUL10, 10 mg/kg) and GW6471 (GW, 1 mg/kg) on the number of GFAP positive cells induced by HI in the CA3 area of the hippocampus and the somatosensory cortex. **(A,C)** In the CA3, a significant effect of lesion [*F*(1,51) = 40.3, *p* < 0.001], treatment [*F*(1,51) = 17.8, *p* < 0.001], and significant interactions between antagonist and treatment [*F*(1,51) = 21.0, *p* < 0.001], and between treatment and lesion [*F*(1,51) = 30.2, *p* < 0.001] were observed. ^
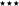
^*p* < 0.001 (lesion effect) ^###^*p* < 0.001 (treatment effect). **(B,D)** In the somatosensory cortex, significant effects of lesion [*F*(1,51) = 146.7, *p* < 0.001], and interactions between antagonist and treatment [*F*(1,51)14.8, *p* < 0.001], and between antagonist, treatment and lesion [*F*(1,51) = 14.8, *p* < 0.001] were observed. The data represent mean + SEM cells per mm^2^ in VEH/VEH (*n* = 10 HI mice; *n* = 7 sham-operated), VEH/SUL10 (*n* = 9 HI mice; *n* = 8 sham-operated), GW/VEH (*n* = 6 HI mice; *n* = 7 sham-operated), GW/SUL10 (*n* = 5 HI mice; *n* = 7 sham-operated) treatment groups. The panels show representative images of GFAP expression in CA3 **(C)** and somatosensory cortex **(D)**. Scale bar = 100 μm. ^
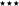
^*p* < 0.001 vs. sham-operated mice of the same group; ^
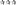
^*p* < 0.001 vs. VEH/VEH HI mice; ^##^*p* < 0.01, ^###^*p* < 0.001 vs. VEH/SUL10 HI mice.

In the CA3 area (**Figures 6A,C**), a significant effect of lesion [*F*(1,51) = 40.3, *p* < 0.001], treatment [*F*(1,51) = 17.8, *p* < 0.001], and significant interactions between antagonist and treatment [*F*(1,51) = 21.0, *p* < 0.001], and treatment and lesion [*F*(1,51) = 30.2, *p* < 0.001] were observed.

In the somatosensory cortex (**Figures 6B,D**), a significant increase in the expression of GFAP was observed in HI mice treated with VEH/VEH (*p* < 0.001), VEH/SUL10 (*p* < 0.001), GW/VEH (*p* < 0.001), and GW/SUL10 (*p* < 0.001) with respect to sham-operated mice. SUL10 significantly reversed the increase in astrocyte expression observed in HI mice treated with VEH (*p* < 0.001), and GW significantly blocked this effect (*p* < 0.01).

In the entorhinal cortex (Supplementary Figures S3A,C), a significant effect of lesion [*F*(1,51) = 825.1, *p* < 0.001], treatment [*F*(1,51) = 107.8, *p* < 0.001], and significant interaction between treatment and lesion [*F*(1,51) = 110.2, *p* < 0.001] were observed. In the piriform cortex (Supplementary Figures S3B,D) a significant effect of lesion [*F*(1,51) = 96.9, *p* < 0.01] was revealed.

### SUL Prevents Neuronal Cell Loss Induced by HI Through the PPAR-α

**FIGURE 7:**
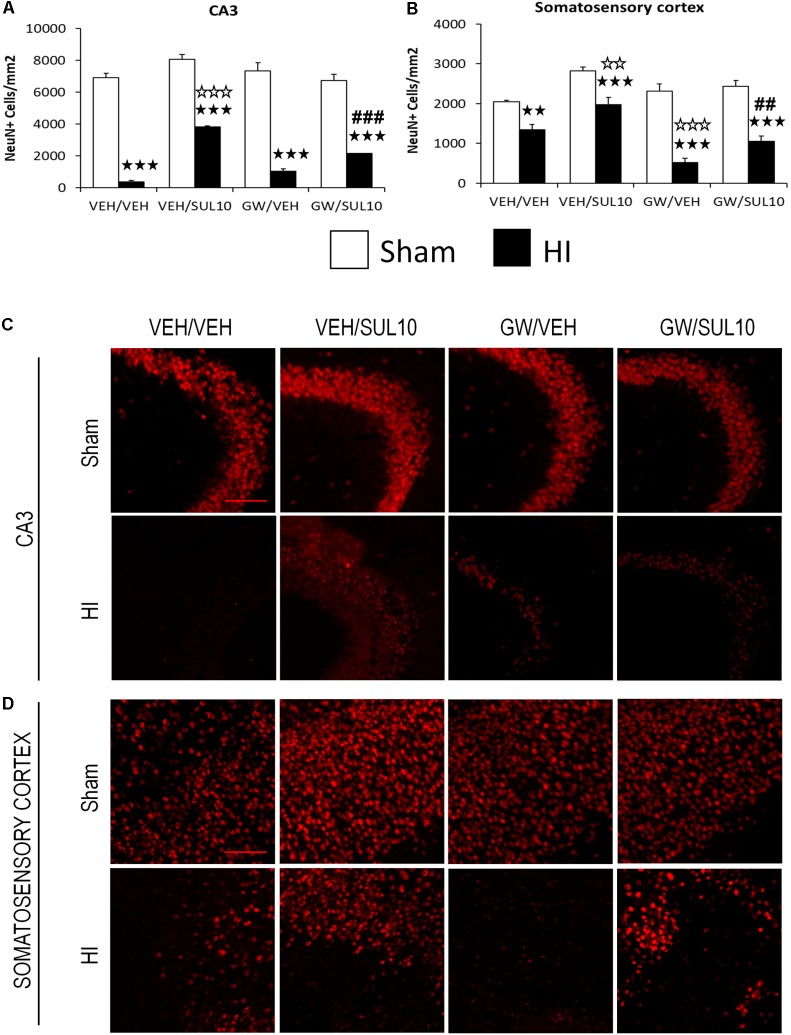
Effects of an acute administration of octadecylpropyl sulfamide (SUL10, 10 mg/kg) and GW6471 (GW, 1 mg/kg) on the number of NeuN positive cells induced by HI. **(A)** In the CA3, significant effects of antagonist [*F*(1,54) = 6.1, *p* < 0.05], treatment [*F*(1,54) = 44.5, *p* < 0.001] and lesion [*F*(1,54) = 818.3, *p* < 0.001], and significant interactions between antagonist and treatment [*F*(1,54) = 28.8, *p* < 0.001], and between treatment and lesion [*F*(1,54) = 28.0, *p* < 0.001] were revealed. **(B)** In the somatosensory cortex, significant effects of antagonist [*F*(1,54) = 12.8, *p* < 0.001], treatment [*F*(1,54) = 29.4, *p* < 0.001], lesion [*F*(1,54) = 104.2, *p* < 0.001], and significant interactions between antagonist and lesion [*F*(1,54) = 9.4, *p* < 0.01], and antagonist, treatment and lesion [*F*(1,54) = 3.7, *p* < 0.05] were observed. Data represent mean + SEM cells per mm^2^ in VEH/VEH (*n* = 11 HI mice; *n* = 7 sham-operated), VEH/SUL10 (*n* = 9 HI mice; *n* = 8 sham-operated), GW/VEH (*n* = 7 HI mice; *n* = 7 sham-operated), GW/SUL10 (*n* = 5 HI mice; *n* = 8 sham-operated) treatment groups. The panels show representative images of NeuN expression in the ipsilateral of CA3 **(C)** and somatosensory cortex **(D)**. Scale bar = 100 μm. ^
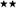
^*p* < 0.01, ^
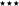
^*p* < 0.001 vs. sham-operated mice of the same group; ^
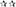
^*p* < 0.01, ^
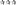
^*p* < 0.001 vs. VEH/VEH HI mice; ^##^*p* < 0.01, ^###^*p* < 0.001 vs. VEH/SUL10 HI mice.

In the CA3 (**Figures 7A,C**), the expression of NeuN decreased in HI mice treated with VEH/VEH (*p* < 0.001), VEH/SUL10 (*p* < 0.001), GW/VEH (*p* < 0.001), and GW/SUL10 (*p* < 0.001) with respect to sham-operated animals. SUL10 significantly reversed the decrease in NeuN expression observed in HI mice treated with VEH (*p* < 0.001), and GW significantly blocked this effect (*p* < 0.001).

In the somatosensory cortex (**Figures 7B,D**), the expression of NeuN was also decreased in HI mice treated with VEH/VEH (*p* < 0.01), VEH/SUL10 (*p* < 0.001), GW/VEH (*p* < 0.001), and GW/SUL10 (*p* < 0.001) with respect to sham-operated animals. SUL10 significantly reversed the decrease in NeuN expression observed in HI mice treated with VEH (*p* < 0.01), and GW significantly blocked this effect (*p* < 0.01).

In the rest of structures studied, only simple effects were revealed: dentate gyrus (Supplementary Figures S4A,C) significant effects of antagonist [*F*(1,54) = 4.9, *p* < 0.05], and lesion [*F*(1,54) = 27.6, *p* < 0.001]; CA1 (Supplementary Figures S4B,D) significant effect of lesion [*F*(1,54) = 10.0, *p* < 0.01]; entorhinal cortex (Supplementary Figures S5A,C) significant effects of treatment [*F*(1,54) = 5.9, *p* < 0.05] and lesion [*F*(1,54) = 9.2, *p* < 0.01]; piriform cortex (Supplementary Figures S5B,D) significant effect of lesion [*F*(1,54) = 14.8, *p* < 0.001].

### Changes Induced by HI in the Expression of Genes Associated With the *N*-Acylethanolamides/Endocannabinoid Signaling Systems

**FIGURE 8:**
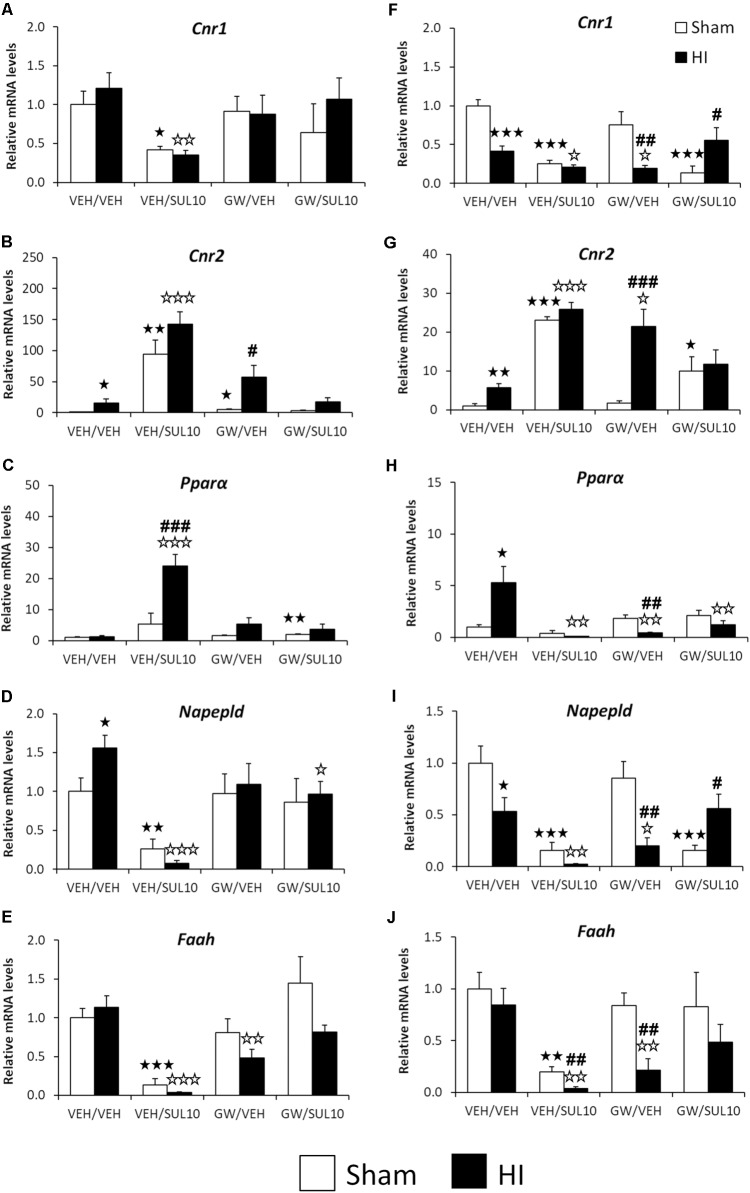
Effects of an acute administration of octadecylpropyl sulfamide (SUL10, 10 mg/kg) and GW6471 (GW, 1 mg/kg) following HI on the gene expression levels of components of the NAEs/endocannabinoid systems *Cnr1* **(A)**, *Cnr2* **(B)**, *Ppar*α **(C)**, *Napepld* **(D),** and *Faah* **(E)** in the ipsilateral motor cortex, and *Cnr1* **(F)**, *Cnr2* **(G)**, *Ppar*α **(H)**, *Napepld* **(I)**, and *Faah* **(J)** in the ipsilateral hippocampus. All data expressed as mean + SEM (*n* = 6). ^
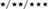
^*p* < 0.05/0.01/0.001 vs. VEH/VEH sham group; ^
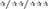
^*p* < 0.05/0.01/0.001 vs. VEH/VEH HI group; ^#/##/###^*p* < 0.05/0.01/0.001 vs. respective VEH/SUL10, GW/VEH and/or GW/SUL10-treated sham group.

Significant changes in the expression of genes related to the NAEs/endocannabinoid systems were observed in the ipsilateral motor cortex (see Supplementary Table S1 for ANOVA values). *Post hoc* analysis indicated that HI increased the gene expression levels of *Cnr2* (**Figure 8B**) and *Napepld* (**Figure 8D**) in mice treated with VEH with respect to those of sham-operated mice with similar treatment (*p* < 0.05). SUL treatment induced a further increase in the gene expression of *Cnr2* (**Figure 8B**) and a decrease in the levels of *Cnr1*, *Napepld*, and *Faah* (**Figures 8A,D,E**) of both sham (*p* < 0.05, *p* < 0.01, *p* < 0.001, respectively) and HI mice (*p* < 0.01, *p* < 0.001, *p* < 0.001, respectively) with respect to those of mice treated with vehicle. Moreover, SUL increased *Ppar*α mRNA levels in HI mice compared to those of sham mice (*p* < 0.001) and HI mice treated with vehicle (*p* < 0.001) (**Figure 8C**). Lesioned mice treated with GW only showed increased *Cnr2* mRNA levels compared to those of sham mice treated with GW (*p* < 0.05) (**Figure 8B**). HI mice treated with GW showed reduced *Faah* mRNA levels compared to those of vehicle-treated HI mice (*p* < 0.01) (**Figure 8E**). Finally, co-administration of SUL and GW in HI mice decreased the *Napepld* mRNA levels as compared to those of HI mice treated with vehicle (*p* < 0.05) (**Figure 8D**).

Significant changes in the expression of genes related to the NAEs/endocannabinoid systems were also observed in the ipsilateral hippocampus (see Supplementary Table S1 for ANOVA values). *Post hoc* analysis indicated that HI increased the mRNA levels of *Cnr2* (*p* < 0.01) and *Ppar*α (*p* < 0.05) (**Figures 8G,H**), and decreased the expression levels of *Cnr1* (*p* < 0.001) and *Napepld* (*p* < 0.05) (**Figures 8F,I**). No changes in the *Faah* mRNA levels were observed in ischemic mice (**Figure 8J**). As described in the motor cortex, SUL treatment also increased the gene expression of *Cnr2* in sham (*p* < 0.001) and lesioned mice (*p* < 0.001) (**Figure 8G**), and decreased expression levels of *Cnr1*, *Napepld*, and *Faah* (**Figures 8F,I,J**) in both sham (*p* < 0.001, *p* < 0.001, *p* < 0.01, respectively) and HI (*p* < 0.05, *p* < 0.01, *p* < 0.01, respectively) mice as compared to the respective vehicle groups. Moreover, HI mice treated with SUL exhibited a higher decrease in *Faah* mRNA levels compared to those of sham mice with similar treatment (*p* < 0.01) (**Figure 8J**). In contrast to motor cortex, *Ppar*α mRNA levels were decreased to a greater extent in HI mice with respect to those of sham mice both treated with SUL (*p* < 0.01) (**Figure 8H**). GW treatment increased *Cnr2* mRNA levels of HI mice compared to those of sham (*p* < 0.001) (**Figure 8G**) and HI mice treated with vehicle (*p* < 0.05) (**Figure 8G**). A decrease in the mRNA levels of *Cnr1* (*p* < 0.05), *Napepld* (*p* < 0.05), and *Faah* (*p* < 0.01) was observed in GW-treated HI mice, but not sham mice, when compared to the respective vehicle HI groups and to the respective GW-treated sham mice (*p* < 0.01) (**Figures 8F,I,J**). By contrast to motor cortex, *Ppar*α mRNA levels were specifically decreased in GW-treated HI mice (*p* < 0.01) (**Figure 8H**). Co-administration of SUL and GW in sham mice increased *Cnr2* gene expression (*p* < 0.05) (**Figure 8G**), and decreased expression levels of *Cnr1* and *Napepld* (*p* < 0.001) (**Figures 8F,I**). These differences were evident when compared to the same group of HI mice (*p* < 0.05) (**Figures 8F,I**). By contrast, co-administration only produced a decrease in *Ppar*α mRNA levels in HI mice (*p* < 0.01) (**Figure 8H**).

### Changes Induced by HI in the Expression of Genes Associated With the Neuroimmune System

**FIGURE 9:**
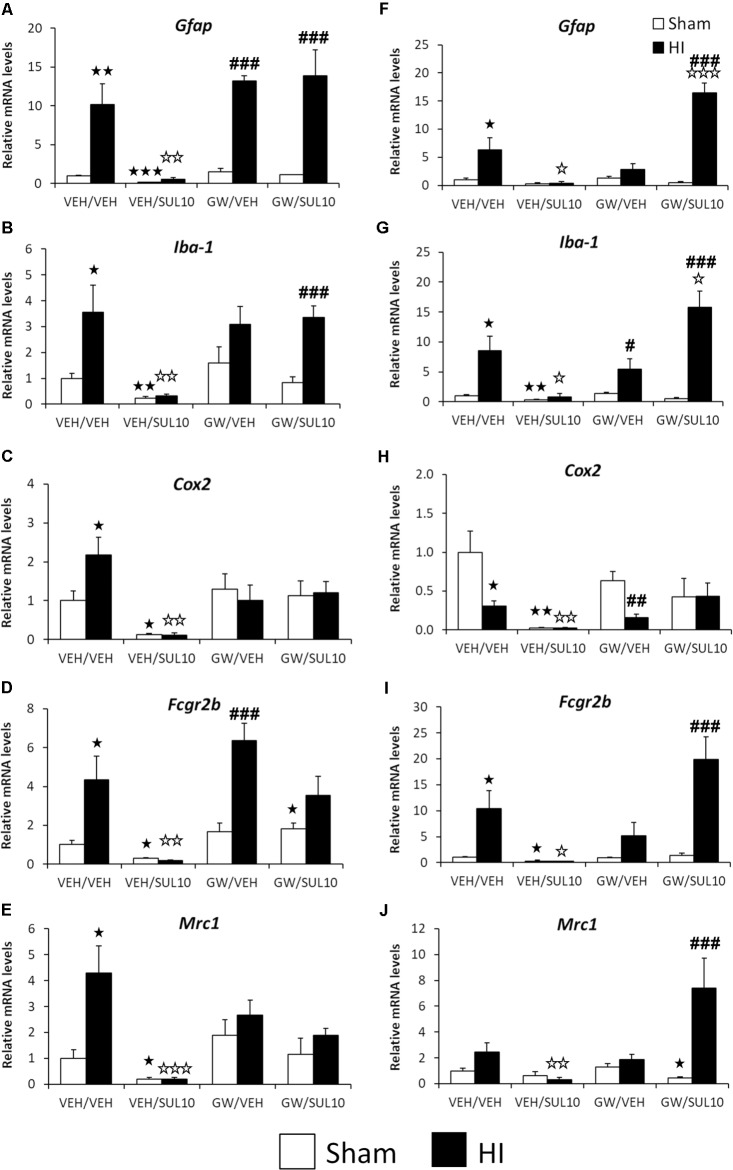
Effects of an acute administration of octadecylpropyl sulfamide (SUL10, 10 mg/kg) and GW6471 (GW, 1 mg/kg) following HI on the gene expression levels of elements of the neuroimmune system *Gfap* **(A)***, Iba-1* **(B)**, *Cox-2* **(C)**, *Fcgr2b* **(D)**, and *Mrc1* **(E)** in the ipsilateral motor cortex, and *Gfap* **(F)***, Iba-1* **(G)**, *Cox-2* **(H)**, *Fcgr2b* **(I)**, and *Mrc1* **(J)** in the ipsilateral hippocampus. All data expressed as mean + SEM (*n* = 6). ^
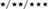
^*p* < 0.05/0.01/0.001 vs. VEH/VEH sham group; ^
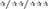
^*p* < 0.05/0.01/0.001 vs. VEH/VEH HI group; ^#/##/###^*p* < 0.05/0.01/0.001 vs. respective VEH/SUL10, GW/VEH and/or GW/SUL10-treated sham group.

In order to clarify the mechanisms of the HI-induced inflammatory reaction associated with microglial activation, the gene expression of several inflammatory markers was measured. Significant changes in the expression of genes related to the neuroimmune system were observed in the ipsilateral motor cortex (see Supplementary Table S2 for ANOVA values). *Post hoc* analysis indicated that HI increased the gene expression of *Gfap*, *Iba-1*, *Cox2*, *Fcgr2b*, and *Mrc1* (**Figures 9A–E**), suggestive of both M1 and M2-type microglial activation, as well as reactive astrocytosis. Interestingly, SUL treatment decreased the gene expression levels of all these factors analyzed in both sham (*p* < 0.05–*p* < 0.01) and HI (*p* < 0.01–*p* < 0.001) mice compared to those of the respective vehicle-treated mice (**Figures 9A–E**).

The GW treatment increased *Gfap* and *Fcgr2b* mRNA levels in HI mice compared to those of GW-treated sham mice (*p* < 0.001) (**Figures 9A,D**). Similarly, co-administration of both drugs in HI mice increased the gene expression of *Gfap* and *Iba-1* compared to the GW/SUL-treated sham mice (*p* < 0.001) (**Figures 9A,B**) suggestive of pharmacological antagonism of the beneficial actions of SUL on inflammatory markers. Co-administration of SUL and GW did not produce remarkable changes in the mRNA levels in the motor cortices of the sham mice (**Figures 9A–E**).

Significant changes in the expression of genes related to HI-induced neuroinflammation were also observed in the ipsilateral hippocampus (see Supplementary Table S2 for ANOVA values). *Post hoc* analysis indicated that HI increased the gene expression of *Gfap*, *Iba-1* and *Fcgr2b* in lesioned mice (*p* < 0.05) (**Figures 9F,G,I**). In contrast to motor cortex, the ipsilateral hippocampus of HI mice showed decreased mRNA levels of *Cox2* (*p* < 0.05) (**Figure 9H**). SUL treatment decreased the gene expression of all factors analyzed in HI mice (**Figures 9F–J**). Regarding sham mice, a decrease in the mRNA levels of *Iba-1*, *Cox2*, and *Fcgr2b* was detected (*p* < 0.05–*p* < 0.01) (**Figures 9G–I**). GW treatment decreased the gene expression of *Cox2* and increased the expression of *Iba-1* of HI mice, compared to the GW-treated sham mice (*p* < 0.05, *p* < 0.01, respectively) (**Figures 9G,H**). No effects on gene expression were observed after GW treatment in sham mice. Interestingly, after co-administration of SUL and GW, HI mice increased the gene expression of *Gfap* (*p* < 0.05) and *Iba-1* (*p* < 0.001) when they were compared to vehicle-treated HI mice and to sham mice with similar treatment (*p* < 0.001) (**Figures 9F,G**). Significant differences in the mRNA levels of *Fcgr2b* and *Mrc1* were also observed in HI mice when co-treated with SUL and GW with respect to sham mice treated with SUL+GW (*p* < 0.001) (**Figures 9I,J**).

## Discussion

The major finding in this study was that SUL reversed the cognitive alterations and motor coordination deficits observed in a mouse model of HI by reducing neuronal loss and microglia overexpression in the hippocampus and cortex. In addition, SUL normalized the changes in the expression of genes associated with HI-induced neuroinflammatory response in these brain structures, especially those of the NAEs/endocannabinoid signaling systems, and those related to activation of astrocytes and microglial phenotypes M1 and M2.

As previously reported (Kossatz et al., 2016), the mouse model of HI used in this study induced motor and cognitive deficits consistent with the extensive damage observed in the striatum, the hippocampus, the cortex and the amygdala, brain structures that are closely associated with these processes (Wang and Morris, 2010; Sasaki et al., 2015). This model resembles that of hypoxic/ischemic stroke in humans, where a marked release of NAEs has been described (Schabitz et al., 2002). Thus, a microdialysis study in stroke patients revealed a peak of anandamide associated to glutamate release in the early phases, followed by a major peak of OEA, a natural PPAR-α activator, that has anti-inflammatory and protective roles (Schabitz et al., 2002). Following these findings, the exogenous administration of a potent and stable OEA analog like SUL was expected to be advantageous after HI. Accordingly, we found that a single post-HI dose of SUL (10 mg/kg) totally reversed the observed memory deficits by acting on the PPRA-α, since the specific antagonist, GW blocked these beneficial effects. Several studies have suggested an important role of the PPAR-α in learning and memory, and this nuclear receptor is abundantly distributed in different areas involved in cognitive processing, such as the cortex, hippocampus and amygdala (Moreno et al., 2004). In a previous study performed in another ischemia model consisting of a medial carotid artery occlusion (MCAO), chronic treatment with the PPRA-α ligand OEA also reduced the cognitive deficits and promoted neurogenesis, as well as neuroplasticity in the ischemic hippocampus (Yang et al., 2015). There is also evidence regarding the involvement of PPAR-α activation in the recovery of memory impairments and hippocampal neurogenesis after global cerebral ischemia (Xuan et al., 2015). Moreover, immunolocalization studies suggest that this nuclear receptor might play a role in regulating the expression of genes associated with cholinergic neurotransmission. Likewise, SUL also reversed the coordination and balance deficits observed following HI, and GW prevented this action. Very few studies have evaluated the effects of PPRA-α agonists on motor impairments induced by cerebral ischemia. One study shows that activation of PPRA-α by fenofibrates induced motor improvements in the grip strength test and prevented neurologic deficits induced in rats and mice subjected to MCAO (Ouk et al., 2014). To investigate the mechanisms involved in the beneficial effects of SUL, we first examined the consequences of an acute post-HI administration on neuroprotection 7 days after brain injury. Remarkably, SUL reduced neuronal cell loss induced by HI in the ipsilateral hippocampus and somatosensory cortex, and that effect was blocked by the PPAR-α antagonist GW. In line with this result, previous studies have shown that SUL (Rodríguez de Fonseca et al., 2012) and OEA (Galan-Rodriguez et al., 2009) increase cellular viability in an *in vitro* model of 6-OHDA-induced degeneration of dopamine neurons. Additionally, SUL also reduced the overexpression of reactive astrocytes, and the increase in microglia induced by HI in the hippocampus, entorhinal, piriform and somatosensory cortices. Astrocytes are thought to promote brain repair following traumatic brain injury and stroke, and are up-regulated under HI conditions in the ipsilateral cerebral cortex of mice (Olson and McKeon, 2004; Koh et al., 2015). Microglia, the resident immune cells in the brain, are downregulated in the healthy normal brain. However, they are susceptible to any imbalance in the CNS homeostasis, and become activated during most neuropathological conditions, such as neurodegenerative diseases and stroke (Liu and Hong, 2003). The inflammatory response in these pathological processes is mediated by the activated microglia, which normally reacts against neuronal injury eliminating the damaged cells by phagocytosis (Dheen et al., 2007), an effect produced when microglia cells are in the M1 phenotype. In parallel, proliferation of microglial cells to an M2 phenotype occurs to provide neurotrophic support through the release of anti-inflammatory cytokines and trophic factors (Tang and Le, 2016). However, persistent inflammation through and M1/M2 imbalance could lead to detrimental consequences instead of the expected beneficial effects. Thus, the chronic activation of microglia may also cause neuronal damage through the release of pro-inflammatory cytokines, reactive oxygen intermediates and other cytotoxic molecules, which collaboratively contribute to ischemic brain injury (Jin et al., 2010). Consequently, suppression of microglia-mediated inflammation has been considered as a useful approach in the therapy of different neuropathological conditions (Dheen et al., 2007).

Our results suggest that SUL regulates inflammatory processes in the hippocampus and cortex in order to limit the detrimental action of HI in the brain by inhibiting the activation/proliferation of microglial phenotypes. In line with this finding, we observed that SUL normalized the expression of genes related to excitotoxicity, neuroinflammation and apoptosis, such as *Gfap*, *Iba-1*, and *Fcgr2b* in the hippocampus. Similar results were observed in the motor cortex, where SUL normalized the expression of *Gfap*, *Iba-1*, *Cox2, Fcgr2b*, and *Mrc1*. Thus, SUL could prevent cognitive and motor impairments following HI by normalizing the cellular and biochemical responses leading to cell loss and inflammation. The fact that GW, a specific PPAR-α antagonist, reversed this response indicates that the beneficial effects of SUL stem from the well-known potential anti-inflammatory actions of PPAR-α activation, as it has been described in other inflammatory processes, such as ulcerative colitis (Suárez et al., 2012).

The administration of SUL following HI upregulated the *Cnr2* mRNA levels in the hippocampus and motor cortex of both sham and HI mice, suggesting a general modulation of this receptor by PPAR-α-mechanisms. In agreement, previous studies have demonstrated that PPAR-α can upregulate CB2 receptors (CB2R) in vascular models of inflammation, facilitating endocannabinoid-mediated vascular protection (Xu et al., 2016). In mice with brain damage following HI, the upregulation of this receptor induced by SUL may be acting to reduce inflammation. Indeed, in a previous study performed in our laboratory, we showed that CB2R play an important role in neuroprotection, acting as a defensive mechanism to prevent neuroinflammation mediated by HIF-1α and TIM-3 in microglia (Kossatz et al., 2016). Several studies have reported that CB2R expression is increased in different models of ischemia, such as MCAO and HI (Ashton et al., 2007; Zhang et al., 2008). Interestingly, a decrease in *Cnr2* mRNA expression was initially reported after transient MCAO, but a marked increase was observed 24 h after treatment, suggesting that CB2R expression increases later in the time course of the ischemia (Zhang et al., 2008). This delayed increase is consistent with the presence of these receptors on macrophages, leukocytes and microglia that are recruited into the ischemic brain in the secondary phase following ischemia (Heinel et al., 1994; Maresz et al., 2005). Activation of CB2R by endocannabinoids during HI might help to limit the extension of the inflammatory response and to promote repair. In addition, the observed decrease in the expression of *Faah*, the main degradation enzyme for anandamide and NAEs, induced by the administration of SUL may extend the benefits of the enhanced NAEs’ response by impairing their degradation. It is important to remark that sulfamoyl derivatives of OEA are not substrates of FAAH, nor inhibitors of its activity (Cano et al., 2007). Thus, the consistent inhibitor effects of SUL observed in *Faah* gene expression are pharmacologically dependent on SUL-induced activation of PPAR-α receptors.

In contrast to *Cnr2* mRNA, our results showed a decrease in *Cnr1* gene expression in hippocampus induced by HI, but no effect in motor cortex. There are controversial results regarding the expression levels in CB1 in stroke models. Some studies have reported an increase in *Cnr1* expression in models of transient MCAO (Jin et al., 2000; Zhang et al., 2008). By contrast, studies in the hippocampus revealed no effect on CB1 receptor (CB1R) binding site density in a model of global ischemia (Schomacher et al., 2006) or in immunohistochemical expression of CB1R in a model of perinatal asphyxia (Blanco et al., 2015). Interestingly, a study performed in a model of ischemia tolerance induction in gerbils revealed that a short ischemic period was associated with a decrease in CB1R protein or binding site density in the hippocampus (Schomacher et al., 2006). The exposure of gerbils to a short period of global ischemia reduces neuronal loss in response to a subsequent, longer ischemic episode, suggesting that downregulation of CB1R may contribute in endogenous post-ischemic neuroprotection (Hillard, 2008). Controversial results have also been reported in non-ischemic models of brain injury (Hansen et al., 2001). Although in our study *Cnr1* gene expression was only decreased in the hippocampus following HI, the administration of SUL reduced its expression in both the hippocampus and the motor cortex. The hippocampus is particularly vulnerable to ischemic insults (Nikonenko et al., 2009), and we found that HI induced a prominent injury in this structure, while less damage was observed in the motor cortex.

The expression of *Napepld* mRNA, the primary enzyme that catalyzes the release of NAEs, was decreased in the hippocampus, contrasting with the increased levels found in the motor cortex. This decrease in *Napepld* levels is consistent with a previous study performed in a model of perinatal asphyxia (Blanco et al., 2015). However, another study did not show changes in *Napepld* mRNA levels after permanent MCAO (Degn et al., 2007). Although *Napepld* gene expression was modulated by HI in a distinctive manner in the hippocampus and motor cortex, the administration of SUL reduced its expression in both structures, an effect similar to that found in *Cnr1*. A plausible explanation may stand on the anti-inflammatory nature of *Napepld* products generated in macrophages and potentially in microglia through a PPAR-α dependent mechanism (Pontis et al., 2016). Exogenous administration of a PPAR-α agonist might decrease the release of NAEs through feedback mechanisms. However, this hypothesis needs to be confirmed. The area-dependent actions of SUL on *Ppar*α expression might reflect a differential distribution of existing PPAR-α receptors and the interference of additional modulators of its expression. Upon activation of PPAR-α, this receptor is translocated to the nucleus, where it can also induce its own expression. Therefore, an activation or feedback suppression of its mRNA expression could be detected depending on the moment of the analysis. Further investigation is needed to understand this differential regulation regarding the brain area studied.

The PPAR-α antagonist, GW induced several significant effects when administered with VEH specifically in HI-lesioned mice. Because of the neuroprotective role of PPAR-α activation under HI conditions (Sun et al., 2007; Zhou et al., 2012, 2017; Yang et al., 2015), it was expected that the inhibitory action of GW would potentiate the detrimental effects of HI on the different behavioral, biochemical and genetic outcomes evaluated in our study. Accordingly, GW/VEH potentiated the decrease in NeuN immunostaining observed in HI-lesioned mice with respect to VEH/VEH administration in the somatosensory cortex, and selectively decreased this parameter in the dentate gyrus and CA1 area of the hippocampus in HI mice, indicating a greater neurotoxic effect. In behavioral tests, however, GW/VEH only potentiated the coordination and balance deficits observe 24 h following HI, while it did not modulate any other behavioral alterations observed in lesioned mice. Unexpectedly, GW/VEH did not increase the expression of pro-inflammatory genes in the cortex or the hippocampus when compared to VEH/VEH treatment, although it induced several specific effects in HI-lesioned compared to sham-operated mice. Thus, GW/VEH treatment selectively increased the mRNA levels of *Gfap, Fcgr2b* in the motor cortex, and in the hippocampus it increased *Iba-1*, and decreased *Cox2* mRNA levels in HI-lesioned mice. This specific effect of GW in mice subjected to HI injury was also evident for genes related to the endocannabinoid system, especially in the hippocampus, where the expression of *Cnr2*, *Cnr1*, *Ppara, Napepld*, and *Faah* mRNA was modulated specifically by GW in lesioned mice, and not in sham-operated controls. These findings suggest that blockade of PPAR-α by GW induces changes in the expression of several genes related to the neuroimmune/endocannabinoid signaling systems following HI especially in brain areas showing large lesions like the hippocampus.

## Conclusion

The SUL may reverse the cognitive and motor coordination impairments induced by HI through the activation of PPAR-α receptors, enhancing *Cnr2*, decreasing *Faah* and downregulating *Cnr1* gene expression leading to a reduction of inflammatory parameters and neuronal cell loss in the hippocampus and cortex. Multiple efforts are in progress to target the inflammatory pathways with the main objective of reducing their detrimental effect on the brain after the ischemic injury. Currently, many research studies have been conducted to investigate the effect of different anti-inflammatory treatments after ischemic stroke. Most of them reported worse outcomes or no differences (Tobin et al., 2014; Bhalala et al., 2015), indicating that more investigation is needed to find new strategies of treatment against the inflammatory process. The activation of the PPAR-α has been described as an important mediator in neuroprotection, and an interesting target for the treatment of brain injury. In the brain, this receptor is involved in the suppression of glial neuroinflammatory responses, and the modulation of other processes, such as ROS metabolism, neuroprotection against oxidative stress, cell proliferation and death (Fidaleo et al., 2014). The potent and stable PPAR-α agonist SUL appears to be a good candidate to counteract the harmful effect of HI brain damage, especially in restoring the cognitive and motor function.

## Author Contributions

EK, DS-P, and JS performed the experiments and quantified the data. EK, JS, FF, RM, and PR designed the experiments, analyzed and discussed the data, and contributed to manuscript writing.

## Conflict of Interest Statement

The authors declare that the research was conducted in the absence of any commercial or financial relationships that could be construed as a potential conflict of interest.

## References

[B1] Ashton J. C. Rahman R. M. Nair S. M. Sutherland B. A. Glass M. Appleton I. (2007). Cerebral hypoxia-ischemia and middle cerebral artery occlusion induce expression of the cannabinoid CB2 receptor in the brain. *Neurosci. Lett.* 412 114–117. 10.1016/j.neulet.2006.10.053 17123706

[B2] Bhalala U. S. Koehler R. C. Kannan S. (2015). Neuroinflammation and neuroimmune dysregulation after acute hypoxic-ischemic injury of developing brain. *Front. Pediatr.* 2:144. 10.3389/fped.2014.00144 25642419 PMC4294124

[B3] Blanco E. Galeano P. Holubiec M. I. Romero J. I. Logica T. Rivera P. (2015). Perinatal asphyxia results in altered expression of the hippocampal acylethanolamide/endocannabinoid signaling system associated to memory impairments in postweaned rats. *Front. Neuroanat.* 9:141. 10.3389/fnana.2015.00141 26578900 PMC4630311

[B4] Blanquart C. Barbier O. Fruchart J. C. Staels B. Glineur C. (2003). Peroxisome proliferator-activated receptors: regulation of transcriptional activities and roles in inflammation. *J. Steroid Biochem. Mol. Biol.* 85 267–273. 10.1016/S0960-0760(03)00214-0 12943712

[B5] Cano C. Pavon J. Serrano A. Goya P. Paez J. A. de Fonseca F. R. (2007). Novel sulfamide analogs of oleoylethanolamide showing in vivo satiety inducing actions and PPARα activation. *J. Med. Chem.* 50 389–393. 10.1021/jm0601102 17228882

[B6] Cutando L. Busquets-Garcia A. Puighermanal E. Gomis-González M. Delgado-García J. M. Gruart A. (2013). Microglial activation underlies cerebellar deficits produced by repeated cannabis exposure. *J. Clin. Invest.* 123 2816–2831. 10.1172/JCI67569 23934130 PMC3696568

[B7] Degn M. Lambertsen K. L. Petersen G. Meldgaard M. Artmann A. Clausen B. H. (2007). Changes in brain levels of N-acylethanolamines and 2-arachidonoylglycerol in focal cerebral ischemia in mice. *J. Neurochem.* 103 1907–1916. 10.1111/j.1471-4159.2007.04892.x 17868306

[B8] Dheen S. T. Kaur C. Ling E.-A. (2007). Microglial activation and its implications in the brain diseases. *Curr. Med. Chem.* 14 1189–1197. 10.2174/092986707780597961 17504139

[B9] Duval C. Chinetti G. Trottein F. Fruchart J.-C. Staels B. (2002). The role of PPARs in atherosclerosis. *Trends Mol. Med.* 8 422–430. 10.1016/S1471-4914(02)02385-7 12223313

[B10] Fidaleo M. Fanelli F. Ceru M. Moreno S. (2014). Neuroprotective properties of peroxisome proliferator-activated receptor alpha (PPARα) and its lipid ligands. *Curr. Med. Chem.* 21 2803–2821. 10.2174/0929867321666140303143455 24606520

[B11] Flynn R. W. V. MacWalter R. S. M. Doney A. S. F. (2008). The cost of cerebral ischaemia. *Neuropharmacology* 55 250–256. 10.1016/j.neuropharm.2008.05.031 18573263

[B12] Galan-Rodriguez B. Suarez J. Gonzalez-Aparicio R. Bermudez-Silva F. J. Maldonado R. Robledo P. (2009). Oleoylethanolamide exerts partial and dose-dependent neuroprotection of substantia nigra dopamine neurons. *Neuropharmacology* 56 653–664. 10.1016/j.neuropharm.2008.11.006 19070629

[B13] Hansen H. H. Schmid P. C. Bittigau P. Lastres-Becker I. Berrendero F. Manzanares J. (2001). Anandamide, but not 2-arachidonoylglycerol, accumulates during in vivo neurodegeneration. *J. Neurochem.* 78 1415–1427. 10.1046/j.1471-4159.2001.00542.x 11579150

[B14] Heinel L. A. Rubin S. Rosenwasser R. H. Vasthare U. S. Tuma R. F. (1994). Leukocyte involvement in cerebral infarct generation after ischemia and reperfusion. *Brain Res. Bull.* 34 137–141. 10.1016/0361-9230(94)90010-8 8044688

[B15] Hillard C. J. (2008). Role of cannabinoids and endocannabinoids in cerebral ischemia. *Curr. Pharm. Des.* 14 2347–2361. 10.2174/138161208785740054 18781985 PMC2581413

[B16] Irwin S. (1968). Comprehensive observational assessment: Ia. A systematic, quantitative procedure for assessing the behavioral and physiologic state of the mouse. *Psychopharmacologia* 13 222–257. 10.1007/BF00401402 5679627

[B17] Jin K. L. Mao X. O. Goldsmith P. C. Greenberg D. A. (2000). CB1 cannabinoid receptor induction in experimental stroke. *Ann. Neurol.* 48 257–261. 10.1002/1531-8249(200008)48:2<257::AID-ANA18>3.0.CO;2-P 10939579

[B18] Jin R. Yang G. Li G. (2010). Inflammatory mechanisms in ischemic stroke: role of inflammatory cells. *J. Leukoc. Biol.* 87 779–789. 10.1189/jlb.1109766 20130219 PMC2858674

[B19] Koh H. S. Chang C. Y. Jeon S.-B. Yoon H. J. Ahn Y.-H. Kim H.-S. (2015). The HIF-1/glial TIM-3 axis controls inflammation-associated brain damage under hypoxia. *Nat. Commun.* 6:6340. 10.1038/ncomms7340 25790768 PMC4383004

[B20] Kossatz E. Maldonado R. Robledo P. (2016). CB2 cannabinoid receptors modulate HIF-1α and TIM-3 expression in a hypoxia-ischemia mouse model. *Eur. Neuropsychopharmacol.* 26 1972–1988. 10.1016/j.euroneuro.2016.10.003 28253997

[B21] Lee C.-H. Olson P. Evans R. M. (2003). Minireview: lipid metabolism, metabolic diseases, and peroxisome proliferator-activated receptors. *Endocrinology* 144 2201–2207. 10.1210/en.2003-0288 12746275

[B22] Levine S. (1960). Anoxic-ischemic encephalopathy in rats. *Am. J. Pathol.* 36 1–17. 14416289 PMC1942188

[B23] Liu B. Hong J.-S. (2003). Role of microglia in inflammation-mediated neurodegenerative diseases: mechanisms and strategies for therapeutic intervention. *J. Pharmacol. Exp. Ther.* 304 1–7. 10.1124/jpet.102.035048 12490568

[B24] Lo E. H. Dalkara T. Moskowitz M. A. (2003). Mechanisms, challenges and opportunities in stroke. *Nat. Rev. Neurosci.* 4 399–415. 10.1038/nrn1106 12728267

[B25] Maresz K. Carrier E. J. Ponomarev E. D. Hillard C. J. Dittel B. N. (2005). Modulation of the cannabinoid CB2 receptor in microglial cells in response to inflammatory stimuli. *J. Neurochem.* 95 437–445. 10.1111/j.1471-4159.2005.03380.x 16086683

[B26] Marx N. Duez H. Fruchart J.-C. Staels B. (2004). Peroxisome proliferator-activated receptors and atherogenesis: regulators of gene expression in vascular cells. *Circ. Res.* 94 1168–1178. 10.1161/01.RES.0000127122.22685.0A 15142970

[B27] Mathers C. D. Loncar D. Boreham J. Thun M. Heath J. Doll R. (2006). Projections of global mortality and burden of disease from 2002 to 2030. *PLoS Med.* 3:e442. 10.1371/journal.pmed.0030442 17132052 PMC1664601

[B28] Moreno S. Farioli-vecchioli S. Cerù M. P. (2004). Immunolocalization of peroxisome proliferator-activated receptors and retinoid X receptors in the adult rat CNS. *Neuroscience* 123 131–145. 10.1016/j.neuroscience.2003.08.064 14667448

[B29] Moreno-Santos I. Pavón F. J. Romero-Cuevas M. Serrano A. Cano C. Suardíaz M. (2014). Computational and biological evaluation of N-octadecyl-N’-propylsulfamide, a selective PPARα agonist structurally related to N-acylethanolamines. *PLoS One* 9:e92195. 10.1371/journal.pone.0092195 24651609 PMC3961330

[B30] Nikonenko A. G. Radenovic L. Andjus P. R. Skibo G. G. (2009). Structural features of ischemic damage in the hippocampus. *Anat. Rec.* 292 1914–1921. 10.1002/ar.20969 19943345

[B31] Olson E. E. McKeon R. J. (2004). Characterization of cellular and neurological damage following unilateral hypoxia/ischemia. *J. Neurol. Sci.* 227 7–19. 10.1016/j.jns.2004.07.021 15546586

[B32] Ouk T. Gautier S. Pétrault M. Montaigne D. Maréchal X. Masse I. (2014). Effects of the PPAR-α agonist fenofibrate on acute and short-term consequences of brain ischemia. *J. Cereb. Blood Flow Metab.* 34 542–551. 10.1038/jcbfm.2013.233 24398933 PMC3948136

[B33] Paxinos G. Franklin K. B. J. (2001). *The Mouse Brain in Stereotaxic Coordinates*, 2nd Edn. San Diego: Academic Press.

[B34] Pontis S. Ribeiro A. Sasso O. Piomelli D. (2016). Macrophage-derived lipid agonists of PPAR-α as intrinsic controllers of inflammation. *Crit. Rev. Biochem. Mol. Biol.* 51 7–14. 10.3109/10409238.2015.1092944 26585314

[B35] Ramírez-López M. T. Arco R. Decara J. Vázquez M. Rivera P. Blanco R. N. (2016). Long-term effects of prenatal exposure to undernutrition on cannabinoid receptor-related behaviors: sex and tissue-specific alterations in the mRNA expression of cannabinoid receptors and lipid metabolic regulators. *Front. Behav. Neurosci.* 10:241. 10.3389/fnbeh.2016.00241 28082878 PMC5187359

[B36] Rodríguez de Fonseca F. Suárez Pérez J. Romero Cuevas M. Fernández Espejo E. Goya Laza M. P. Páez Prosper J. A. (2012). *Use of Sulfamide Derivatives as Neuroprotectors.* Available at: https://patentscope.wipo.int/search/en/detail.jsf?docId=WO2012131142&recNum=1&maxRec=&office=&prevFilter=&sortOption=&queryString=&tab=PCT$+$Biblio

[B37] Rousselet E. Kriz J. Seidah N. G. (2012). Mouse model of intraluminal MCAO: cerebral infarct evaluation by cresyl violet staining. *J. Vis. Exp.* 69:4038. 10.3791/4038 23168377 PMC3520579

[B38] Sasaki T. Leutgeb S. Leutgeb J. K. (2015). Spatial and memory circuits in the medial entorhinal cortex. *Curr. Opin. Neurobiol.* 32 16–23. 10.1016/j.conb.2014.10.008 25463560 PMC4416067

[B39] Schabitz W.-R. Giuffrida A. Berger C. Aschoff A. Schwaninger M. Schwab S. (2002). Release of fatty acid amides in a patient with hemispheric stroke: a microdialysis study. *Stroke* 33 2112–2114. 10.1161/01.STR.0000023491.63693.18 12154273

[B40] Schomacher M. Müller H. D. Sommer C. (2006). Short-term ischemia usually used for ischemic preconditioning down-regulates central cannabinoid receptors in the gerbil hippocampus. *Acta Neuropathol.* 111 8–14. 10.1007/s00401-005-1109-2 16328514

[B41] Suárez J. Romero-Zerbo Y. Márquez L. Rivera P. Iglesias M. Bermúdez-Silva F. J. (2012). Ulcerative colitis impairs the acylethanolamide-based anti-inflammatory system reversal by 5-aminosalicylic acid and glucocorticoids. *PLoS One* 7:e37729. 10.1371/journal.pone.0037729 22662201 PMC3360619

[B42] Sun Y. Alexander S. P. H. Garle M. J. Gibson C. L. Hewitt K. Murphy S. P. (2007). Cannabinoid activation of PPAR alpha; a novel neuroprotective mechanism. *Br. J. Pharmacol.* 152 734–743. 10.1038/sj.bjp.0707478 17906680 PMC2190030

[B43] Tang Y. Le W. (2016). Differential roles of M1 and M2 microglia in neurodegenerative diseases. *Mol. Neurobiol.* 53 1181–1194. 10.1007/s12035-014-9070-5 25598354

[B44] Tobin M. K. Bonds J. A. Minshall R. D. Pelligrino D. A. Testai F. D. Lazarov O. (2014). Neurogenesis and inflammation after ischemic stroke: what is known and where we go from here. *J. Cereb. Blood Flow Metab.* 34 1573–1584. 10.1038/jcbfm.2014.130 25074747 PMC4269726

[B45] Toyama T. Nakamura H. Harano Y. Yamauchi N. Morita A. Kirishima T. (2004). PPARα ligands activate antioxidant enzymes and suppress hepatic fibrosis in rats. *Biochem. Biophys. Res. Commun.* 324 697–704. 10.1016/j.bbrc.2004.09.110 15474484

[B46] Wang S.-H. Morris R. G. M. (2010). Hippocampal-neocortical interactions in memory formation, consolidation, and reconsolidation. *Annu. Rev. Psychol.* 61 49–79. 10.1146/annurev.psych.093008.100523 19575620

[B47] Xu X. Guo H. Jing Z. Yang L. Chen C. Peng L. (2016). N-oleoylethanolamine reduces inflammatory cytokines and adhesion molecules in tnf-α-induced human umbilical vein endothelial cells by activating CB2 and PPAR-α. *J. Cardiovasc. Pharmacol.* 68 280–291. 10.1097/FJC.0000000000000413 27281236

[B48] Xuan A.-G. Chen Y. Long D.-H. Zhang M. Ji W.-D. Zhang W.-J. (2015). PPARα agonist fenofibrate ameliorates learning and memory deficits in rats following global cerebral ischemia. *Mol. Neurobiol.* 52 601–609. 10.1007/s12035-014-8882-7 25241646

[B49] Yang L. C. Guo H. Zhou H. Suo D. Q. Li W. J. Zhou Y. (2015). Chronic oleoylethanolamide treatment improves spatial cognitive deficits through enhancing hippocampal neurogenesis after transient focal cerebral ischemia. *Biochem. Pharmacol.* 94 257–269. 10.1016/j.bcp.2015.02.012 25748831

[B50] Zhang M. Martin B. R. R. Adler M. W. W. Razdan R. K. K. Ganea D. Tuma R. F. F. (2008). Modulation of the balance between cannabinoid CB1 and CB2 receptor activation during cerebral ischemic/reperfusion injury. *Neuroscience* 152 753–760. 10.1016/j.neuroscience.2008.01.022 18304750 PMC2577828

[B51] Zhou H. Yang W.-S. Li Y. Ren T. Peng L. Guo H. (2017). Oleoylethanolamide attenuates apoptosis by inhibiting the TLR4/NF-κB and ERK1/2 signaling pathways in mice with acute ischemic stroke. *Naunyn Schmiedebergs Arch. Pharmacol.* 390 77–84. 10.1007/s00210-016-1309-4 27738712

[B52] Zhou Y. Yang L. Ma A. Zhang X. Li W. Yang W. (2012). Orally administered oleoylethanolamide protects mice from focal cerebral ischemic injury by activating peroxisome proliferator-activated receptor a. *Neuropharmacology* 63 242–249. 10.1016/j.neuropharm.2012.03.008 22480617

[B53] Zola-Morgan S. Squire L. R. (1993). Neuroanatomy of memory. *Annu. Rev. Neurosci.* 16 547–563. 10.1146/annurev.ne.16.030193.002555 8460903

[B54] Zola S. M. Squire L. R. Teng E. Stefanacci L. Buffalo E. A. Clark R. E. (2000). Impaired recognition memory in monkeys after damage limited to the hippocampal region. *J. Neurosci.* 20 451–463. 10.1523/JNEUROSCI.20-01-00451.2000 10627621 PMC6774137

