# Supplementary material for: Octadecylpropyl Sulfamide Reduces Neurodegeneration and Restores the Memory Deficits Induced by Hypoxia-Ischemia in Mice
Source: Front Pharmacol. 2018 Apr 19;9:376. doi: 10.3389/fphar.2018.00376 (PMC5917089; doi:10.3389/fphar.2018.00376)
Supplement: Supplementary file 1 [file Data_Sheet_1.docx]

Octadecylpropyl Sulfamide Reduces Neurodegeneration and Restores the Memory Deficits Induced by Hypoxia-Ischemia in Mice

Elk Kossatz, Daniel Silva-Peña, Juan Suárez, Fernando Rodríguez de Fonseca,

Rafael Maldonado^*^ and Patricia Robledo^*^

*** Correspondence:** Corresponding Author: [probledo@imim.es](mailto:probledo@imim.es)

**1. Supplementary Figures**

## 2. Supplementary Tables


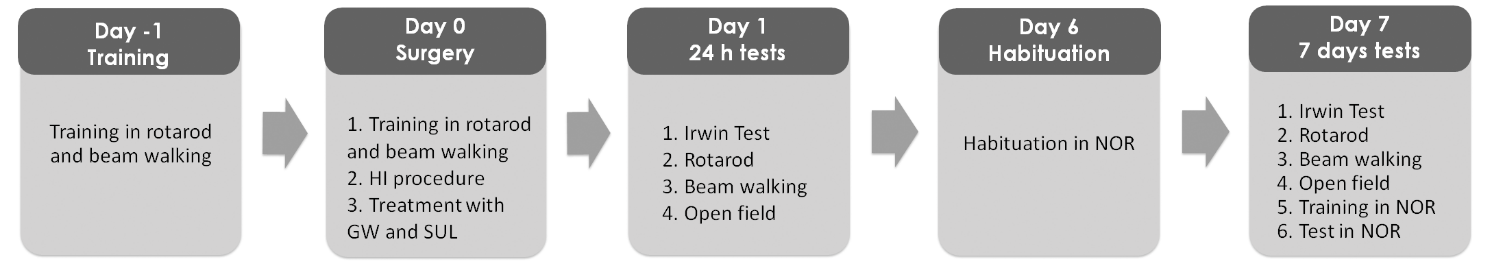


**Supplementary Figure 1.** Time line of behavioral tests 24 h and 7 days after HI procedure. GW: GW6471; SUL: octadecylpropyl sulfamide; NOR: novel object recognition test.


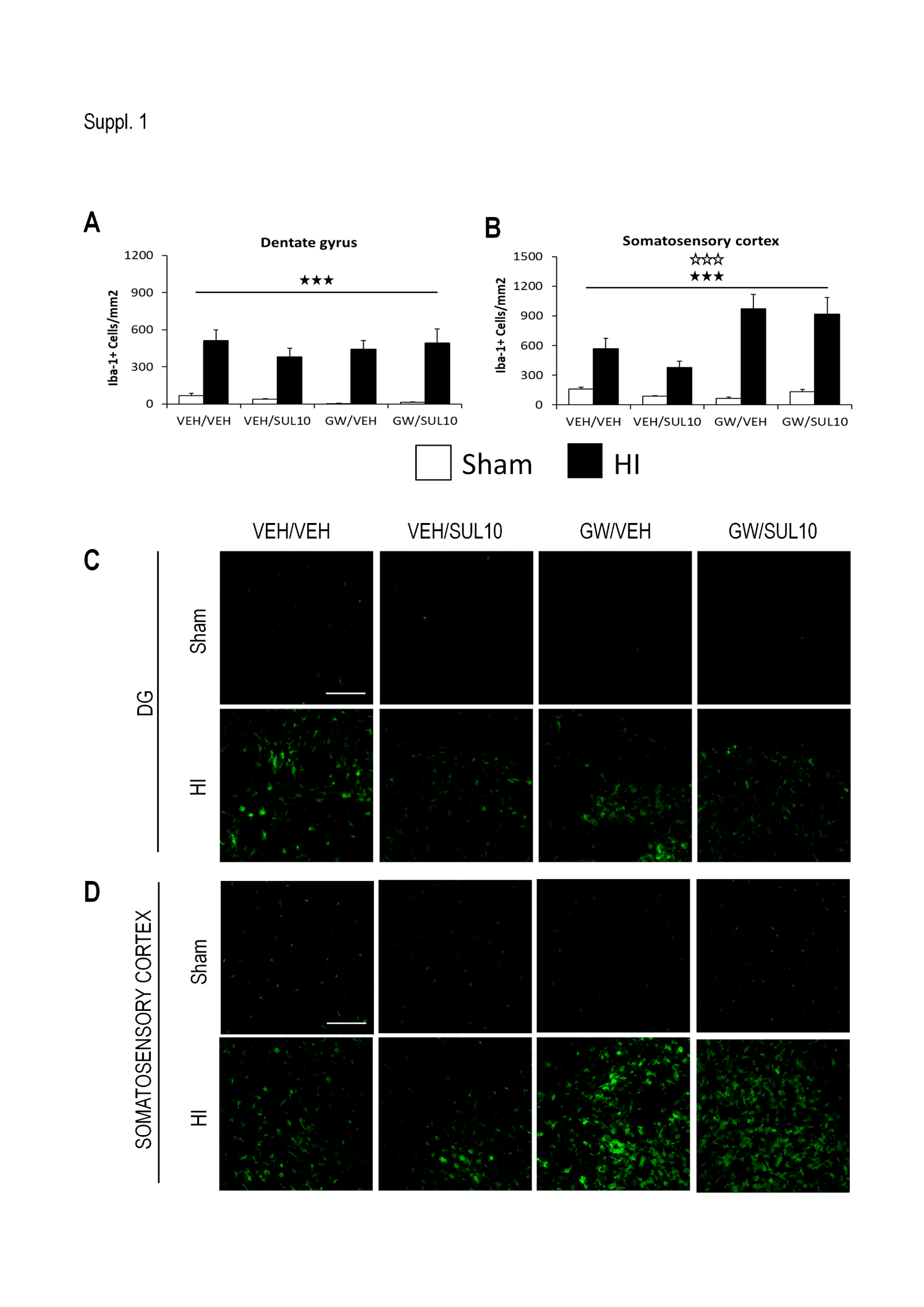


**Supplementary Figure 2.** Effects of an acute administration of octadecylpropyl sulfamide (SUL10, 10 mg/kg) and GW6471 (GW, 1 mg/kg) on the number of Iba-1 positive cells induced by HI in the ipsilateral dentate gyrus (A) and somatosensory cortex (B). Data represent mean +S.E.M. cells per mm^2^ in VEH/VEH (n=11 HI mice; n=7 sham-operated), VEH/SUL10 (n=9 HI mice; n=8 sham-operated), GW/VEH (n=6 HI mice; n=6 sham-operated), GW/SUL10 (n=5 HI mice; n=7 sham-operated) treatment groups. The panels show representative images of Iba-1 expression in dentate gyrus (C) and somatosensory cortex (D). Scale bar = 100 μm. ^★★★^p<0.001 (lesion effect); ^✰✰✰^p<0.001 (antagonist effect).

**
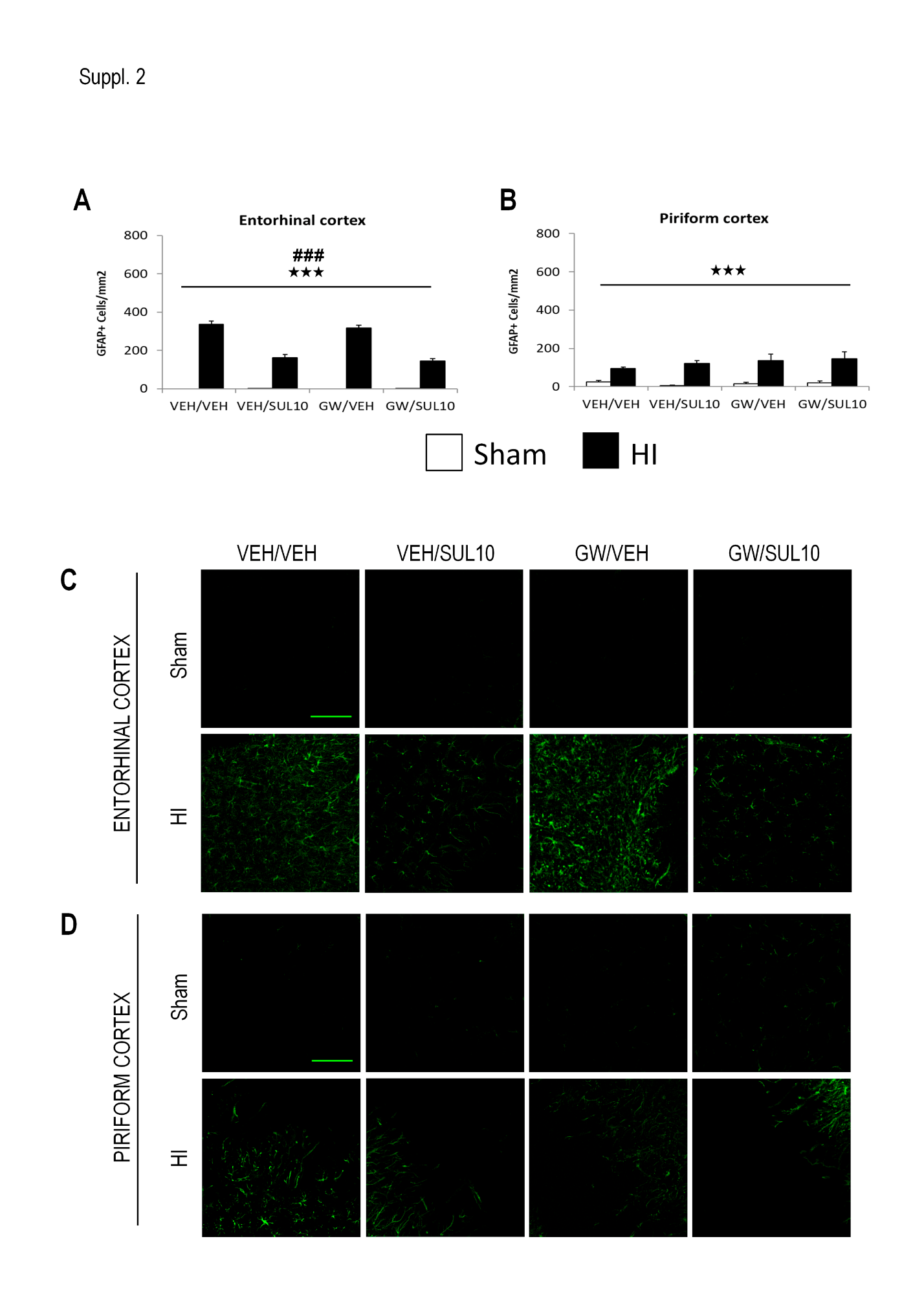
Supplementary Figure 3.** Effects of an acute administration of octadecylpropyl sulfamide (SUL10, 10 mg/kg) and GW6471 (GW, 1 mg/kg) on the number of GFAP positive cells induced by HI in the ipsilateral entorhinal cortex (A) and piriform cortex (B). The data represent mean +S.E.M. cells per mm^2^ in VEH/VEH (n=10 HI mice; n=7 sham-operated), VEH/SUL10 (n=9 HI mice; n=8 sham-operated), GW/VEH (n=6 HI mice; n=7 sham-operated), GW/SUL10 (n=5 HI mice; n=7 sham-operated) treatment groups. The panels show representative images of GFAP expression in the ipsilateral entorhinal cortex (C) and piriform cortex (D). Scale bar = 100 μm. ^★★★^p<0.001 (lesion effect); ^###^p<0.001 (treatment effect).


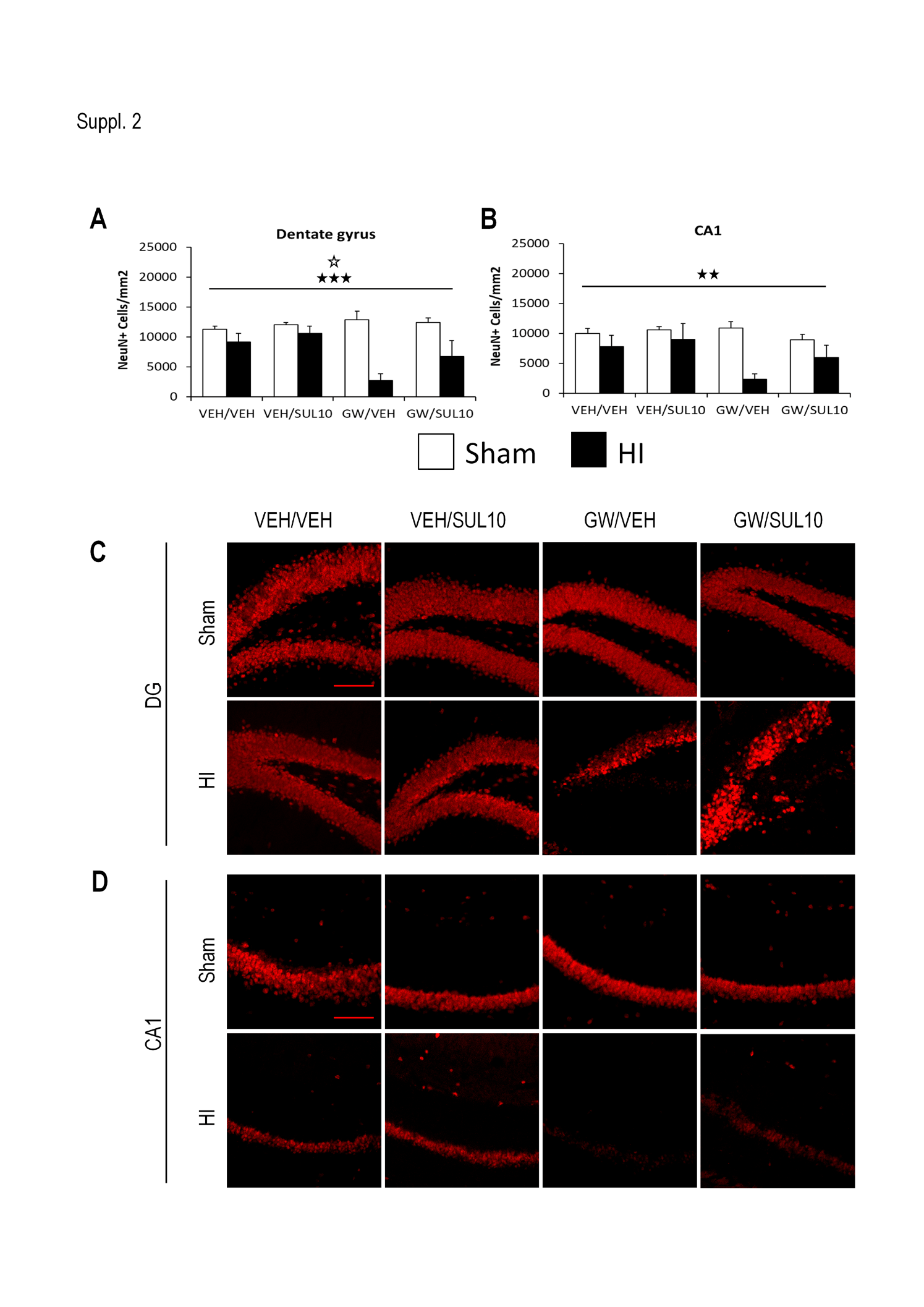
**Supplementary Figure 4.** Effects of an acute administration of octadecylpropyl sulfamide (SUL10, 10 mg/kg) and GW6471 (GW, 1 mg/kg) on the number of NeuN positive cells induced by HI in the ipsilateral hippocampal areas dentate gyrus (A) and CA1 (B). Data represent mean +S.E.M. cells per mm^2^ in VEH/VEH (n=11 HI mice; n=7 sham-operated), VEH/SUL10 (n=9 HI mice; n=8 sham-operated), GW/VEH (n=7 HI mice; n=7 sham-operated), GW/SUL10 (n=5 HI mice; n=8 sham-operated) treatment groups. The panels show representative images of NeuN expression in the ipsilateral dentate gyrus (C) and CA1 (D). Scale bar = 100 μm. ^★★/★★★^p<0.01/0.001 (lesion effect); ^✰^p<0.05 (antagonist effect).


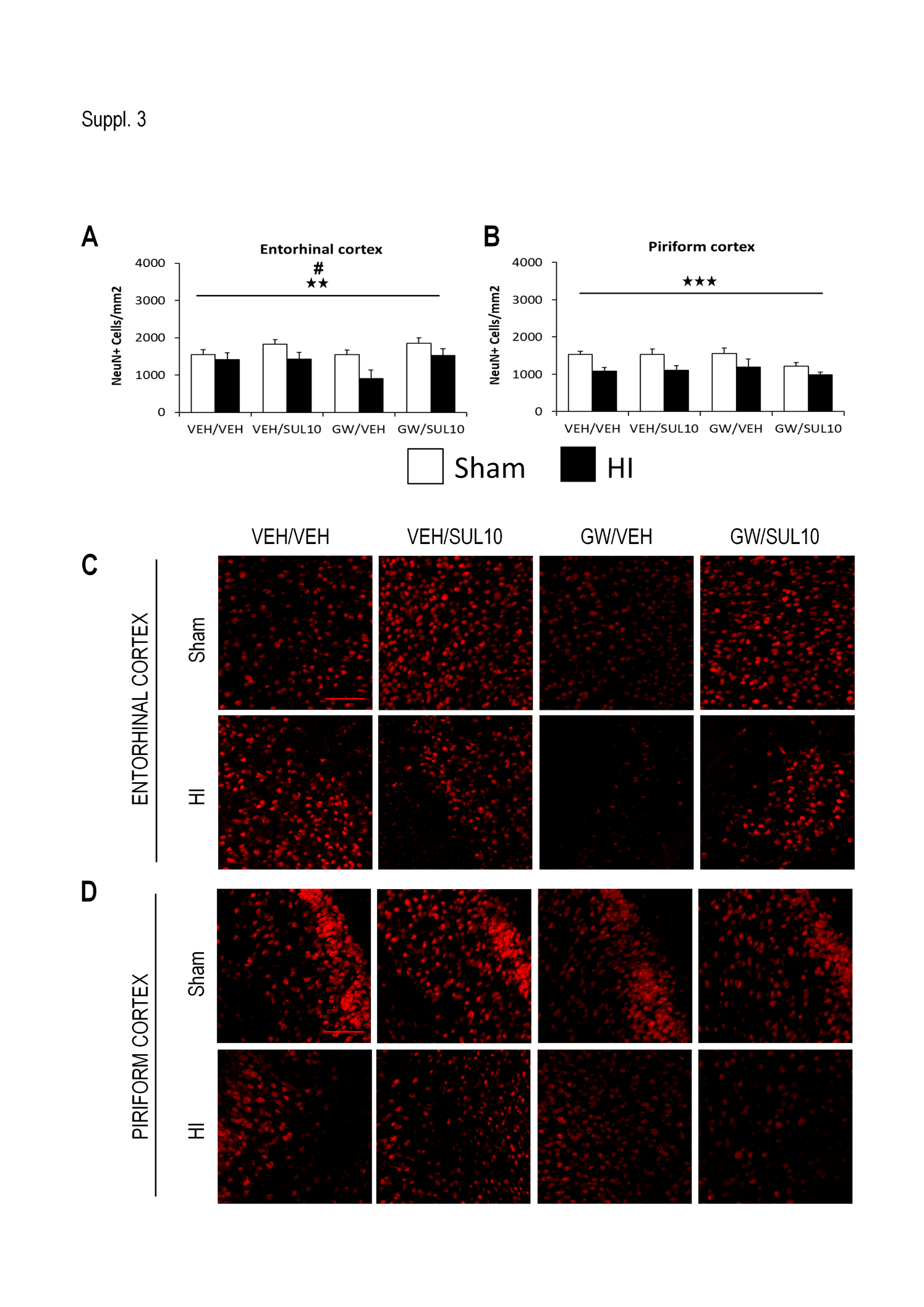


**Supplementary Figure 5.** Effects of an acute administration of octadecylpropyl sulfamide (SUL10, 10 mg/kg) and GW6471 (GW, 1 mg/kg) on the number of NeuN positive cells induced by HI in the ipsilateral entorhinal cortex (A) and piriform cortex (B). Data represent mean +S.E.M. cells per mm^2^ in VEH/VEH (n=11 HI mice; n=7 sham-operated), VEH/SUL10 (n=9 HI mice; n=8 sham-operated), GW/VEH (n=7 HI mice; n=7 sham-operated), GW/SUL10 (n=5 HI mice; n=8 sham-operated) treatment groups. The panels show representative images of NeuN expression in the ipsilateral entorhinal cortex (C) and piriform cortex (D). Scale bar = 100 μm. ^★★/★★★^p<0.01/0.001 (lesion effect); ^#^p<0.05 (treatment effect).

**Supplementary Table 1.** NAEs signaling changes in the ipsilateral cortex and hippocampus. Statistical values for 3-way ANOVAs.

| Gene | Brain structure | Factor | Statistical value |
| --- | --- | --- | --- |
| *Cnr1* | Motor cortex | Treatment | F(1,41)= 7.352, p<0.05 |
|  |  | Treatment-antagonist | F(1,41)= 5.869, p<0.05 |
|  | Hippocampus | Lesion | F(1,41)= 9.807, p<0.01 |
|  |  | Treatment | F(1,41)= 23.808, p<0.001 |
|  |  | Lesion-treatment | F(1,41)= 37.398, p<0.001 |
|  |  | Treatment-antagonist | F(1,41)= 7.854, p<0.01 |
| *Cnr2* | Motor cortex | Lesion | F(1,41)= 15.172, p<0.001 |
|  |  | Treatment | F(1,41)= 28.584, p<0.001 |
|  |  | Antagonist | F(1,41)= 25.821, p<0.001 |
|  |  | Treatment-antagonist | F(1,41)= 61.205, p<0.001 |
|  |  | Lesion-treatment-antagonist | F(1,41)= 4.527, p<0.001 |
|  | Hippocampus | Lesion | F(1,41)= 20.155, p<0.001 |
|  |  | Treatment | F(1,41)= 39.897, p<0.001 |
|  |  | Lesion- treatment | F(1,41)= 9.406, p<0.01 |
|  |  | Lesion-antagonist | F(1,41)= 4.824, p<0.05 |
|  |  | Treatment-antagonist | F(1,41)= 46.102, p<0.001 |
|  |  | Lesion-treatment-antagonist | F(1,41)= 6.123, p<0.05 |
| *Napepld* | Motor cortex | Treatment | F(1,38)= 21.058, p<0.001 |
|  |  | Treatment-antagonist | F(1,38)= 13.660, p<0.01 |
|  | Hippocampus | Lesion | F(1,39)= 7.304, p<0.05 |
|  |  | Treatment | F(1,39)= 29.198, p<0.001 |
|  |  | Lesion-treatment | F(1,39)= 19.816, p<0.001 |
|  |  | Treatment-antagonist | F(1,39)= 10.484, p<0.01 |
|  |  | Lesion-treatment-antagonist | F(1,39)= 5.348, p<0.05 |
| *Faah* | Motor cortex | Lesion | F(1,41)= 4.906, p<0.05 |
|  |  | Treatment | F(1,41)= 5.663, p<0.05 |
|  |  | Antagonist | F(1,41)= 8.957, p<0.01 |
|  |  | Lesion-antagonist | F(1,41)= 5.542, p<0.05 |
|  |  | Treatment-antagonist | F(1,41)= 48.871, p<0.05 |
|  | Hippocampus | Lesion | F(1,41)= 8.875, p<0.01 |
|  |  | Treatment | F(1,41)= 9.933, p<0.01 |
|  |  | Treatment-antagonist | F(1,41)= 18.98, p<0.001 |
| *Pparα* | Motor cortex | Lesion | F(1,34)= 46.306, p<0.001 |
|  |  | Treatment | F(1,34)= 51.559, p<0.001 |
|  |  | Antagonist | F(1,34),= 28.271, p<0.001 |
|  |  | Lesion- treatment | F(1,34)= 20.551, p<0.001 |
|  |  | Lesion-antagonist | F(1,34)= 14.490, p<0.01 |
|  |  | Treatment-antagonist | F(1,34)= 62.16, p<0.001 |
|  |  | Lesion-treatment-antagonist | F(1,34)= 32.82, p<0.001 |
|  | Hippocampus | Treatment | F(1,32)= 7.335, p<0.05 |
|  |  | Lesion- treatment | F(1,32)= 5.505, p<0.05 |
|  |  | Lesion-antagonist | F(1,32)= 12.628, p<0.01 |
|  |  | Treatment-antagonist | F(1,32)= 15.184, p<0.001 |
|  |  | Lesion-treatment-antagonist | F(1,32)= 8.521, p<0.01 |

**Supplementary Table 2.** Neuroimmune signaling changes in the ipsilateral cortex and hippocampus. Statistical values for 3-way ANOVAs.

| Gene | Brain structure | Factor | Statistical value |
| --- | --- | --- | --- |
| *Gfap* | Motor cortex | Lesion | F(1,41)= 67.372, p<0.001 |
|  |  | Treatment | F(1,41)= 6.225, p<0.05 |
|  |  | Antagonist | F(1,41)= 18.599, p<0.001 |
|  |  | Lesion-antagonist | F(1,41)= 12.925, p<0.01 |
|  |  | Treatment-antagonist | F(1,41)= 6.731, p<0.05 |
|  |  | Lesion-treatment-antagonist | F(1,41)= 5.707, p<0.05 |
|  | Hippocampus | Lesion | F(1,41)= 61.076, p<0.001 |
|  |  | Treatment | F(1,41)= 4.409, p<0.05 |
|  |  | Antagonist | F(1,41)= 20.181, p<0.001 |
|  |  | Lesion-treatment | F(1,41)= 9.758, p<0.001 |
|  |  | Lesion-antagonist | F(1,41)= 16.961, p<0.001 |
|  |  | Treatment-antagonist | F(1,41)= 43.972, p<0.001 |
|  |  | Lesion-treatment-antagonist | F(1,41)= 44.854, p<0.001 |
| *Iba-1* | Motor cortex | Lesion | F(1,41)= 19.754, p<0.001 |
|  |  | Treatment | F(1,41)= 8.968, p<0.01 |
|  |  | Antagonist | F(1,41)= 6.411, p<0.05 |
|  |  | Treatment-antagonist | F(1,41)= 5.573, p<0.05 |
|  |  | Lesion-treatment-antagonist | F(1,41)= 5.397, p<0.05 |
|  | Hippocampus | Lesion | F(1,41)= 51.644, p<0.001 |
|  |  | Antagonist | F(1,41)= 10.751, p<0.01 |
|  |  | Lesion-antagonist | F(1,41)= 8.702, p<0.01 |
|  |  | Treatment-antagonist | F(1,41)= 22.242, p<0.001 |
|  |  | Lesion-treatment-antagonist | F(1,41)= 23.005, p<0.001 |
| *Cox2* | Motor cortex | Treatment | F(1,39)= 11.442, p<0.01 |
|  |  | Treatment-antagonist | F(1,39)=11.725, p<0.01 |
|  | Hippocampus | Lesion | F(1,39)=8.194, p<0.01 |
|  |  | Treatment | F(1,39)=8.586, p<0.01 |
|  |  | Lesion-treatment | F(1,39)=8.390, p<0.01 |
|  |  | Treatment-antagonist | F(1,39)=10.512, p<0.01 |
| *Fcgr2b* | Motor cortex | Lesion | F(1,37)= 23.557, p<0.001 |
|  |  | Treatment | F(1,37)= 14.386, p<0.01 |
|  |  | Antagonist | F(1,37)= 14.281, p<0.01 |
|  |  | Lesion-treatment | F(1,37)= 10.44, p<0.01 |
|  | Hippocampus | Lesion | F(1,41)= 29.362, p<0.001 |
|  |  | Antagonist | F(1,41)= 6.911, p<0.05 |
|  |  | Lesion-antagonist | F(1,41)= 5.096, p<0.05 |
|  |  | Treatment-antagonist | F(1,41)= 19.572, p<0.001 |
|  |  | Lesion-treatment-antagonist | F(1,41)= 16.263, p<0.001 |
| *Mrc1* | Motor cortex | Lesion | F(1,38)= 8.378, p<0.01 |
|  |  | Treatment | F(1,38)= 14.983, p<0.001 |
|  |  | Treatment-antagonist | F(1,38)= 4.136, p<0.05 |
|  | Hippocampus | Lesion | F(1,39)= 13.167, p<0.01 |
|  |  | Antagonist | F(1,39)= 7.493, p<0.01 |
|  |  | Lesion-antagonist | F(1,39)= 7.023, p<0.05 |
|  |  | Treatment-antagonist | F(1,39)= 9.022, p<0.01 |
|  |  | Lesion-treatment-antagonist | F(1,39)= 11.545, p<0.01 |
